# Activation of mucosal insulin receptor exacerbates intestinal inflammation by promoting tissue resident memory T cells differentiation through EZH2

**DOI:** 10.1186/s12967-023-04789-x

**Published:** 2024-01-19

**Authors:** Teming Li, Ben Han, Liucan Wang, Lihua Sun, Yujiao Cai, Min Yu, Weidong Xiao, Hua Yang

**Affiliations:** 1 Department of General Surgery, Xinqiao Hospital, Army Medical University, Chongqing, 400037 China; 2 Department of General Surgery, Army 953 Hospital, Shigatse Branch of Xinqiao Hospital, Army Medical University, Shigatse, 857000 China; 3 Department of General Surgery, Chongqing General Hospital, Chongqing, 401147 China

**Keywords:** Intestinal intraepithelial lymphocytes, Insulin receptor, Tissue resident memory T cells, Inflammatory bowel disease

## Abstract

**Background:**

Inflammatory Bowel Diseases (IBD), an autoimmune disease characterised by abnormal intestinal immunity, are related to vital morbidity around the world. However, therapeutic agents for IBD have not achieved desired benefit. Exploring new therapeutic targets for IBD, especially based on its abnormally intestinal immunity, could alleviate the flare-up and worsening of IBD. Tissue resident memory T cells (TRM) are core of multiple autoimmune diseases, including IBD. However, the mechanism of TRM differentiation remains to be investigated.

**Methods:**

The alterations in mRNA and lncRNA profile of intestinal intraepithelial lymphocytes (IELs), the largest component of intestinal TRM, were analyzed in DSS-induced chronic colitis. Based on it, we examined the function of rectal insulin instillation in a dextran sodium sulfate (DSS) induced chronic colitis. Furthermore, we investigated the downstream-target of the insulin pathway—EZH2 and the crucial role of EZH2 in intestinal tissue resident memory T cell differentiation by utilizing EZH2^fl/fl^CD4^cre^ mice.

**Results:**

Insulin receptor (INSR) expression was found to be significantly reduced. Activation of mucosal insulin pathway by rectal insulin instillation exacerbated colitis by disrupting IELs subgroups and up-regulating TNF-ɑ and IL-17 expression. Rectal insulin instillation promoted EZH2 expression and EZH2 inhibition alleviated chronic colitis. EZH2^fl/fl^CD4^cre^ mice restored the normal IEL subgroups and suppressed TNF-ɑ and IL-17 expression, exhibiting alleviated colitis. IELs from EZH2^fl/fl^CD4^cre^ mice exhibit significant changes in TRM related phenotype. CD4^+^TRM was significantly increased in chronic colitis and decreased in EZH2^fl/fl^CD4^cre^ mice.

**Conclusion:**

Insulin receptor of intestinal mucosal T-cells could promote intestinal TRM differentiation via EZH2. Our discoveries suggest that therapies targeting colonic INSR and EZH2 could be potential treatment for IBD based on its regulatory effects on TRM. Insulin receptor inhibitors rather than insulin should be applied during colitis-active phase. In addition, EZH2 shows to be a downstream signal of the insulin pathway and EZH2 inhibitor could alleviating intestinal inflammation. However, the critical role of EZH2 in TRM differentiation restricts the anti-tumor effects of EZH2 inhibitor in vivo.

**Supplementary Information:**

The online version contains supplementary material available at 10.1186/s12967-023-04789-x.

## Background

Inflammatory Bowel Diseases (IBD), represented by Crohn’s Disease (CD) and Ulcerative Colitis (UC), are related to vital morbidity in developed countries and with rapidly growing incidence in the developing world [1]. Uncontrolled activation of intestinal immune cells which inflicting self-destruction is the core of IBD genesis and advancement [2, 3]. Therapeutic agents for IBD include 5-aminosalicylate, glucocorticoids, immunosuppressants, and biological agents, all of which act on different aspects of intestinal immunity. However, about 40% of patients treated with 5-aminosalicylate [4] or glucocorticoids [5] experienced relapse. Even with biological agents (infliximab), more than 30% of patients with severe disease still fail to achieve remission [6]. Therefore, it is of great importance to explore novel IBD pharmaceuticals, especially gut immunity-based pharmaceuticals.

The mechanism below intestinal immune abnormality has not yet been elucidated thoroughly. Although many mucosal immune cells, including T help cells [7], regulatory T cells (Treg) [8], innate lymphoid cells(ILCs) [9], and macrophages [10] are involved in the pathogenesis of IBD, the short life span of most immune cells makes it difficult to explain the persistent immune abnormality in IBD. Generated from effector T cells, tissue resident memory T cells(TRM) characterized by persistent colonization, long life span, self-proliferation, and secretion of inflammatory cytokines, which is extremely compatible with the pathological characteristics of IBD [11]. In recent years, the important role of TRM in IBD has been demonstrated continually [11–13]. Research by Lamb et al. showed that high expression of CD103, one of the intestinal TRM markers, in intestinal CD4^+^T cells was associated with increased production of pro-inflammatory cytokines and decreased expression of immunoregulatory markers in UC patients [14]. Bishu et al. found that CD4^+^TRM is the major source of the inflammatory factor TNF in CD patients [15]. Zundler et al. showed that the core marker of intestinal TRM, CD69 and CD103, was significantly increased in IBD patients, and CD4 TRM was directly related to the recurrence of IBD; TRM depletion alleviates colitis by reducing inflammatory factors and lymphocyte infiltration [12].

As a major component of intestinal TRMs, intestinal intraepithelial lymphocytes (IELs) are the largest population of the intestinal immune system [16]. IELs locate between the basolateral surfaces of the intestinal epithelial cells. Based on its intimate association with intestinal epithelial cells in space, IELs interact closely with intestinal epithelial cells and is essential for the maintenance of gut homeostasis and inflammation [17–19]. Previous studies in IBD have demonstrated that diverse subgroups of IELs exhibit unique effect in intestinal inflammation and IBD. Briefly, CD8αα^+^TCRαβ^+^ and CD8αα^+^TCRγδ^+^ IELs play an anti-inflammatory effect caused by the immune-suppressive effect of CD8aa; CD4^+^ and CD8αβ^+^IELs exerts a pro-inflammatory effect through the secretion of inflammatory factors [16, 20–23]. However, how TRM and IELs are increased and transformed into pathogenic cells in IBD has not been elucidated.

In recent years, expanding studies have shown the critical role of metabolism in the regulation of immunity [24]. The insulin pathway is the master pathway in the regulation of glucose metabolism and also shows a regulatory function in mucosal immunology [25]. High glucose intake, which is tightly controlled by the insulin pathway, exacerbates autoimmunity by inducing Th_17_ cells through ROS-dependent TGF-β activation [26]. Furthermore, insulin receptor signaling reinforces a metabolic program that supports T cell nutrient uptake and associated glycolytic and respiratory capacities [27]. However, insights into the insulin receptor (INSR) as the potential IBD therapeutic target is still lacking.

Multiple mechanisms, including epigenetics, are involved in the metabolic regulation of immunity [28]. Histone modification is an integral component of epigenetics, which could promote/repress gene expression through the regulation of DNA transcription. Enhancer of zeste homolog 2 (EZH2) is an enzymatic catalytic subunit of Polycomb repressive complex 2(PRC2) that can alter gene expression by trimethylation of Lys-27 in histone 3 (H3K27me3) [29, 30], and also could activate downstream genes in a PRC2-independent manner [31, 32]. EZH2 works as a key regulator of T cell differentiation and function, including CD8^+^T cell [33, 34], Treg [35] and follicular helper T cells [36]. However, available studies indicate a paradoxical role for EZH2 in intestinal inflammation. Macrophage/microglial EZH2 facilitates autoimmune inflammation [37]and EZH2 inhibitor GSK343 alleviates experimental colitis by promoting the development of Myeloid-derived suppressor cells [38], indicating a pro-inflammatory effect of EZH2. However, suppressed EZH2 by Fbxw7 in macrophages promoted intestinal inflammation [39], and inactivation of EZH2 in epithelial cells sensitizes mice to experimental colitis, indicating an anti-inflammatory effect of EZH2 [40]. Despite the known positive and negative effects of EZH2 on intestinal inflammation, whether EZH2 exerts its effects during the differentiation of TRM has not been illustrated.

Here, we defined that the INSR of intestinal mucosal T-cells could promote intestinal TRM differentiation via EZH2 and exacerbate chronic colitis. Firstly, this study clarified the changes of IEL-related RNA profiles in DSS-induced chronic colitis. Multiple metabolic pathway alterations were revealed and the expression of INSR was found to be significantly reduced in both intestinal intraepithelial lymphocytes and lamina propria lymphocytes. Second, rectal insulin instillation led to disrupted phenotype and increased inflammatory cytokines in IELs, which could exacerbate colitis. Furthermore, rectal Insulin instillation promotes EZH2 expression and the EZH2 inhibitor GSK126 alleviates colitis. Finally, by using EZH2^fl/fl^CD4^cre^ mice, we demonstrated that EZH2 is essential for the induction of intestinal CD4 and CD8 TRM cells. Disrupted IELs’ subgroup and increased inflammatory cytokines by rectal insulin instillation were resulted from promoted differentiation of colonic TRM through EZH2.

## Material and methods

### Animal and DSS-induced chronic colitis

EZH2^fl/fl^CD4^cre^ were a gift from Dr Lilin Ye (Army Medical University, Chongqing, China) and a total of 5 mice at least per group were used to establish animal models(except BM chimeras). C57BL/6 mice were obtained from the Experiment Animal Center for animal models (Army Medical University, Chongqing, China) and a total of 10 mice per group were used to establish animal models. All procedures in animal models were approved by the University Committee on the Use and Care of Animals of the Army Medical University.

The DSS-induced chronic colitis model was constructed as a protocol published by Neurath [41]. Briefly, mice in the DSS-induced chronic colitis group (DSS group) was treated with 3 cycles of DSS. Each cycle consists of 7 days of drinking water containing 2% DSS and 2 weeks of standard drinking water followed. The Control group used standard drinking water consistently (Additional file 1: Figure S1A). Animal models of DSS + high-glusose, DSS + IPHG and DSS + insulin was constructed as shown in each Figure. In particular, insulin concentration gradients for rectal insulin instillation were set at 2, 6, and 12U/Kg, which based on the inhibitory effect on tumor genesis at 6U/Kg in previous research.

### Isolation and purification of IELs

Intestinal epithelial cell isolation was performed as previously described 39. Briefly, the colon was removed and placed in a tissue culture medium (RPMI 1640, with 2% fetal calf serum). Then, the colon was cut into 5-mm pieces followed by extensively rinsed with ice-cold PBS containing 2% fetal calf serum. The rinsed pieces were then incubated in Ca2^+^- and Mg2^+^-free PBS containing 5 mM EDTA, 2 mM DTT and 10% fetal calf serum for 20 min at 37 °C with continuous brisk stirring. Then, the supernatant was collected and filtered through both 70 and 30 μm MACS SmartStrainers (Miltenyi Biotec) to remove debris and pellets. Intestinal epithelial cells and IELs obtained by centrifugation of collected supernatant can be used directly for flow staining.

After centrifugation, the IELs were purified by 40%/70% Percoll (GE Healthcare Bio-sciences) from Intestinal epithelial cells. Then, the CD3e MicroBead Kit (Miltenyi Biotec) was used to select CD3^+^ intraepithelial lymphocytes according to the manufacturer’s instructions. The purity of the IELs was detected by flow cytometric analysis. The IELs were stained with Bv421 anti-mouse CD3 (BD Biosciences) according to the manufacturer's protocol. The acquisition and analysis were performed using Beckman Coulter Gallios Flow Cytometer (Beckman Coulter).

### Isolation and purification of lamina propria lymphocytes

The lamina propria lymphocytes (LPLs) isolation was performed after the IELs Isolation. Firstly, the intestinal segments undergoing IEL isolation were thoroughly washed with PBS to remove EDTA. The intestinal segments were then putted in a gentle MACS Dissociator (Miltenyi Biotec) and 5 mL of RPMI containing 1 mg/mL collagenaseI was added. The gentle MACS Dissociator is then placed on the gentleMACS Octo Dissociator with Heaters (Miltenyi Biotec). Set the mode to "intestine" and start digestion. After digestion, the supernatant was collected and filtered through both 70 and 30 μm MACS SmartStrainers (Miltenyi Biotec). Lamina propria cells obtained by centrifugation of collected supernatant can be used directly for flow staining. LPLs were purified from Lamina propria cells by 40%/70% Percoll (GE Healthcare Bio-sciences). Purified LPLs were used for qPCR.

### Differentially expressed genes (DEGs) and KEGG pathway analysis

The analysis for DE mRNA and DE lncRNA was performed using R package edgeR. Differentially expressed genes with |log2(FC)| value > 1 and p < 0.05 were considered as significantly modulated and retained for further analysis. This choice is motivated by the decision to maximize the sensitivity of this analysis, in order to perform a massive screening and identify candidate genes to be validated with a wider sample population with real-time PCR analysis. the KEGG pathway analysis (http://www.genome.ad.jp/kegg) were performed via enrich R package. p < 0.05 was considered to be a statistically significant enrichment.

### mRNA and lncRNA joint analysis

To analyze the cis regulation of IncRNAs, the Integrative Genomics Viewer was used to show the genomic loci containing IncRNAs and their adjacent genes. About 100 kb upstream or downstream sequences of IncRNAs were retrieved to search their Candidate mRNA targets. Trans regulation was based on the principle of sequence complementation between mRNA and lncRNA. RNAplex software was used to calculate the thermodynamic parameter value of the complementary pairing, and a parameter value less the − 40 was selected as the target gene of lncRNA.

To identify interactions among the differentially expressed IncRNAs and mRNAs, we constructed a co-expression network based on a Pearson correlation analysis of the differentially expressed IncRNAs and mRNAs. Pearson's correlation coefficients equal to or greater than 0.9 were used to identify the lncRNA and protein-coding genes.

### Flow cytometry and antibodies

Flow cytometry data were obtained with Beckman Coulter Gallios Flow Cytometer (Beckman Coulter) and analyzed using FlowJo software. Surface staining was performed in PBS containing 2% fetal bovine serum (wight/vol) on ice. Intracellular staining of Foxp3, EZH2, IL-17, TNF-α, IFN-γ and Granzyme B was performed using the Foxp3/Transcription Factor Staining Buffer Set (00–5523; eBioscience). Fixable Viability Dye eFluor® (eBioscience) was added to exclude dead cells. The following antibodies were used for flow cytometry: CD3 (BV421, BDbioscience, clone 145-2C11, dilution 1:100), CD69(PE-cy7, BDbioscience, clone H1.2F3, dilution 1:100), CD103 (PE, Biolegend, clone 2E7, dilution 1:100), Klrg1(BV421, BDbioscience, clone 2F1, dilution 1:100), CD25(APC, BDbioscience, clone PC61, dilution 1:100), CD4(APC-cy7,BDbioscience, clone GK1.5, dilution 1:200), CD8a(Percp-cy5.5,BDbioscience, clone 53-6.7, dilution 1:200), ifn-γ (FITC, BDbioscience, clone XMG1.2, dilution 1:50), EZH2 (PE, BDbioscience, clone 11/EZH2, dilution 1:100), CD8a(PE, BDbioscience, clone GK1.5, dilution 1:200), TCRβ (APC, Biolegend, clone 53-6.7, dilution 1:200), CD44(APC, Biolegend, clone IM7, dilution 1:200), CD3 (PE-cy7, Biolegend, clone 17A2, dilution 1:200), TNF-ɑ (FITC, Biolegend, clone MP6-XT22, dilution 1:50), CD8β (FITC, Biolegend, clone YTS156.7.7, dilution 1:100), TCRγδ (FITC, Biolegend, clone GL3, dilution 1:100), GRANZYME B (FITC, Biolegend, clone GB11, dilution 1:100), CXCR6 (PE, Biolegend, clone SA051D1, dilution 1:100).

### Full-thickness colonic tissue culture and CBA

Full-thickness colonic tissue culture was operated as described in previous study [41]. A 1 cm colon was taken from the same colonic region. Remove residual stools by performing three washes with ice-cold PBS containing 20 µg/mL gentamycin. Generate three to four defined circular biopsies with a 3-mm dermal punch tool. Move the individual biopsies to a well of a 48-well plate containing 0.5 mL of sterile RPMI culture medium. Incubate for 12–24 h in a cell culture incubator at 37 °C. Discard the biopsies, and freeze media supernatants at − 20℃ for storage.

BD™ Cytometric Bead Array (CBA) Mouse Th1/Th2/Th17 CBA Kit (BD560485) was used for CBA test and analysis as its guidelines. The concentration of detection sample was determined by the standard curve of the mean fluorescence intensity.

### Bone marrow (BM) chimeras

Bone marrow (BM) chimeras was operated as described in previous study [36]. The femurs of mice were isolated under sterile conditions and the muscle and fibrous tissue from the bones were cut off. Femurs were placed in culture dish and 5–7 mL of RPMI complete medium was added. Cut off both ends of the femur were cut off. Using a 10-mL syringe, 5 mL of RPMI complete medium was aspirated and mounted with a 25-gauge needle. Carefully flush the bone marrow out. The flushing out was repeated until the bone was white. Bone marrow tissue was mashed and passed through a 70 μm MACS SmartStrainers (Miltenyi Biotec). The bone marrow cells were centrifuged and resuspended. A total of 2 × 106 BM cells harvested from Ezh2fl/flCD4-Cre (CD45.2^+^) mice and C57BL/6J wild-type (WT) (CD45.1^+^) mice were mixed at a ratio of 4:6. Mixed BM cells were then intravenously injected into lethally irradiated (two doses of 550 rads each) CD45.1^+^ WT recipients. After 2-month DSS-induced colitis, the colonic TRM were analyzed.

### Statistical analysis

Statistical analyses were conducted with Prism 8.0 software (GraphPad). An unpaired two-tailed t-test with a 95% confidence interval was used to calculate P-values. For BM chimera experiments, and a paired two-tailed t-test with the 95% confidence interval was used to calculate P-values (ns, P > 0.05;*P < 0.05; **P < 0.005; ***P < 0.0005).

## Results

### mRNA profiles of colonic IELs in DSS-induced chronic colitis

DSS-induced colitis is remarkably comparable to ulcerative colitis and is the most commonly used animal models in the studies of IBD. Since IBD is a chronic disease, chronic colitis induced by multiple DSS cycles matches human IBD more closely. RNA-seq analysis was performed previously to identify the dysregulated RNA profile using the whole colon RNA. However, the differential expression can be attenuated or eliminated completely due to the diversity of cell types in the whole colon. The role of the IELs in IBD has not received adequate attention and there have been no studies of IELs related mRNA and long no coding RNA (lncRNA) profiles in colitis or IBD [41]. Thus, we investigated the alteration in mRNA and lncRNA profiles of intestinal intraepithelial lymphocytes (IELs) in the context of DSS**-**induced chronic colitis. A DSS-induced chronic colitis is constructed as shown in Additional file 1: Figure S1. One week after last DSS cycle, intraepithelial lymphocytes (IELs) were isolated from the colons of two groups. A total of 28,673 mRNA and 70,251 lncRNAs in IELs of DSS group and Control group were analyzed by high-throughput sequencing. 6 differentially expressing mRNA (DEmRNA) and 8 differentially expressed lncRNA (DElncRNA) were chosen randomly for verification. The results of qRT-PCR verification were consistent with sequencing results (Additional file 1: Figure S2).

**Fig. 1:**
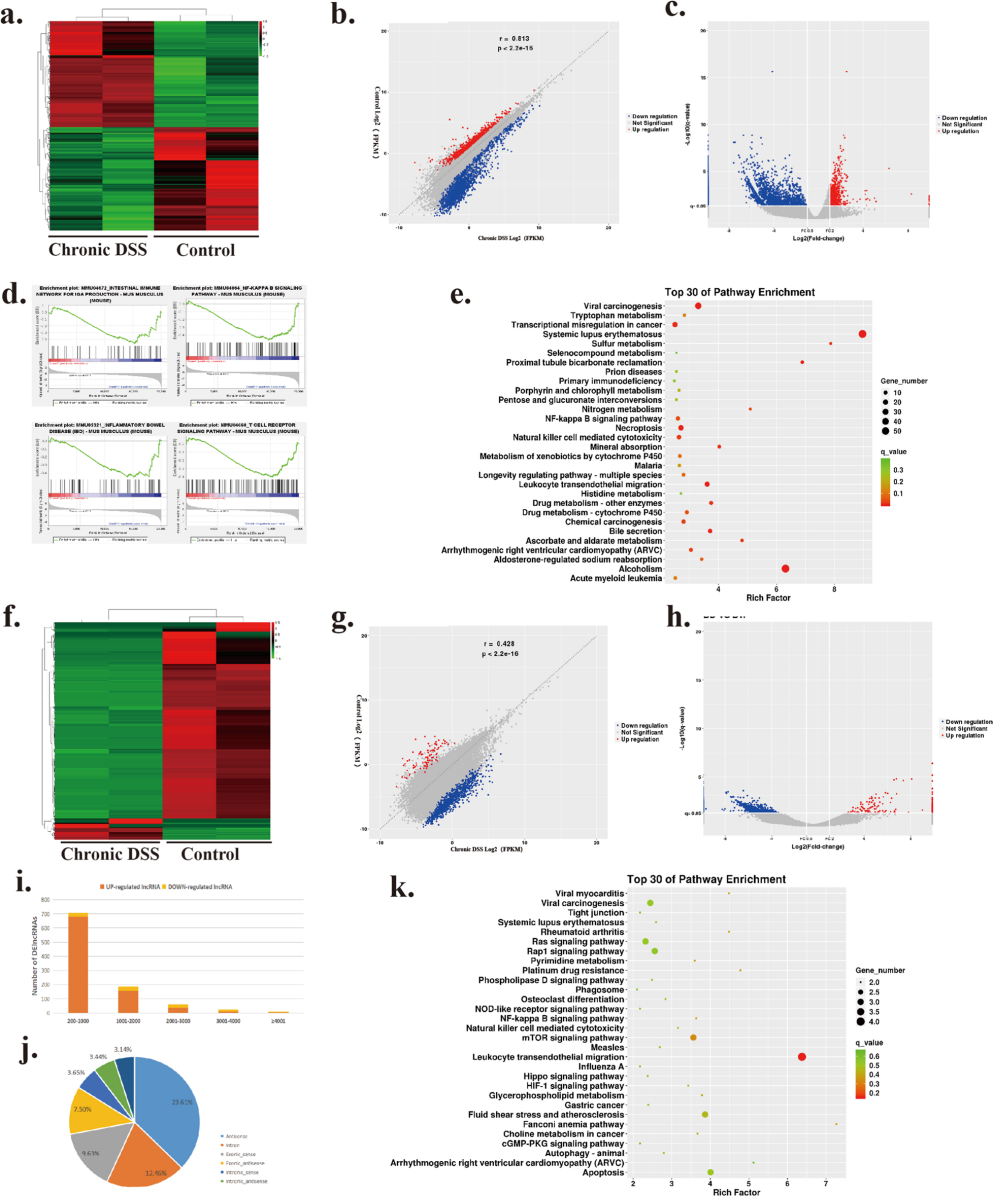
Profiles of DE mRNAs and lncRNAs in DSS induced chronic colitis. **a**–**c** heat map, scatter plot, volcano plot of IELs DEmRNAs expression between the DSS group and the Control group. **d** GSEA analysis of DE mRNAs of Intestinal Immune Network for IgA Prodution, NF-kappa B Signaling Pathway, Inflammatory Bowel Disease and T Cell Receptor Signaling Pathway. e. KEGG pathway analysis of DE mRNAs. **f**–**h** heat map, scatter plot, volcano plot of IELs lncRNAs expression between the DSS group and the Control group. **i** length distribution of the up-regulated and down-regulated lncRNAs. **j** frequency distribution of DE lncRNAs in each lncRNA category. **k** KEGG pathway analysis of DEmRNAs which predicted to be regulated by DElncRNAs

The heat map, scatter plot, volcano plot of mRNA was shown in Fig. 1a–c. KEGG and GSEA pathway analysis was performed on significantly dysregulated mRNAs. As shown in Fig. 1d, GSEA pathway analysis showed that the Inflammatory Bowel Disease, T Cell Receptor Signaling Pathway, NF-kappa B Signaling Pathway, and Intestinal Immune Network for IgA production were enriched in DSS group. As shown in Fig. 1e, the KEGG pathway analysis was performed to show that the DE mRNAs were enriched in NF-kappa B signaling pathway, Natural killer cell mediated cytotoxicity, Leukocyte transendothelial migration, Systemic lupus erythematosus. Among the TOP30 KEGG enrichment pathways, 8 were directly related to metabolism: Tryptophan metabolism, Sulfur metabolism, histidine metabolism, Nitrogen metabolism, Porphyrin and chlorophyll metabolism, ascorbate and aldarate metabolism, pentose and glucuronate interconvenrsions, and Selenocompound metabolism, suggesting a metabolically altered of IELs in chronic colitis.

### Joint analysis of DE mRNA and DE lncRNA

For lncRNAs, the heat map, scatter plot, volcano plot was shown in Fig. 1f–h. In order to detect the expression characteristics of DE lncRNAs, the length distribution of the up-regulated and categories of lncRNAs was analyzed (Fig. 1i, j). DElncRNA and DEmRNA joint analysis were operated to identify interactions among the DElncRNAs and DEmRNAs as described in Material and Methods. The pairs of DElncRNA and its targeting DEmRNA were identified and a KEGG pathway analysis was performed on significant DEmRNAs above (Fig. 1k). KEGG pathway analysis showed that the DEmRNAs have enriched in Leukocyte transendothelial migration, NF-kappa B signaling pathway, Natural killer cell mediated cytotoxicity, mTOR signaling pathway.

### Down-regulation of insulin receptor in DSS-induced chronic colitis

Among the pairs screened by DEmRNA and DElncRNA joint analysis, both INSR and the INSR-regulating lncRNA, MSTRG.175050.1, showed highly significant down-regulated fold changes in colitis (INSR top10, MSTRG.175050.1 top30, Additional file 1: Table S1). A protein–protein interaction and ceRNA network of INSR were shown in Additional file 1: Figure S3. As previously described, IELs is metabolically altered in chronic colitis. Insulin pathway is the master regulator of glucose metabolism [25]. On the other hand, the role of insulin and glucose metabolism in inflammation has received increasing attention in recent years [26, 27, 42]. However, the role and mechanism of intestinal mucosal insulin pathway in colitis and IBD have not been elucidated.

**Fig. 2:**
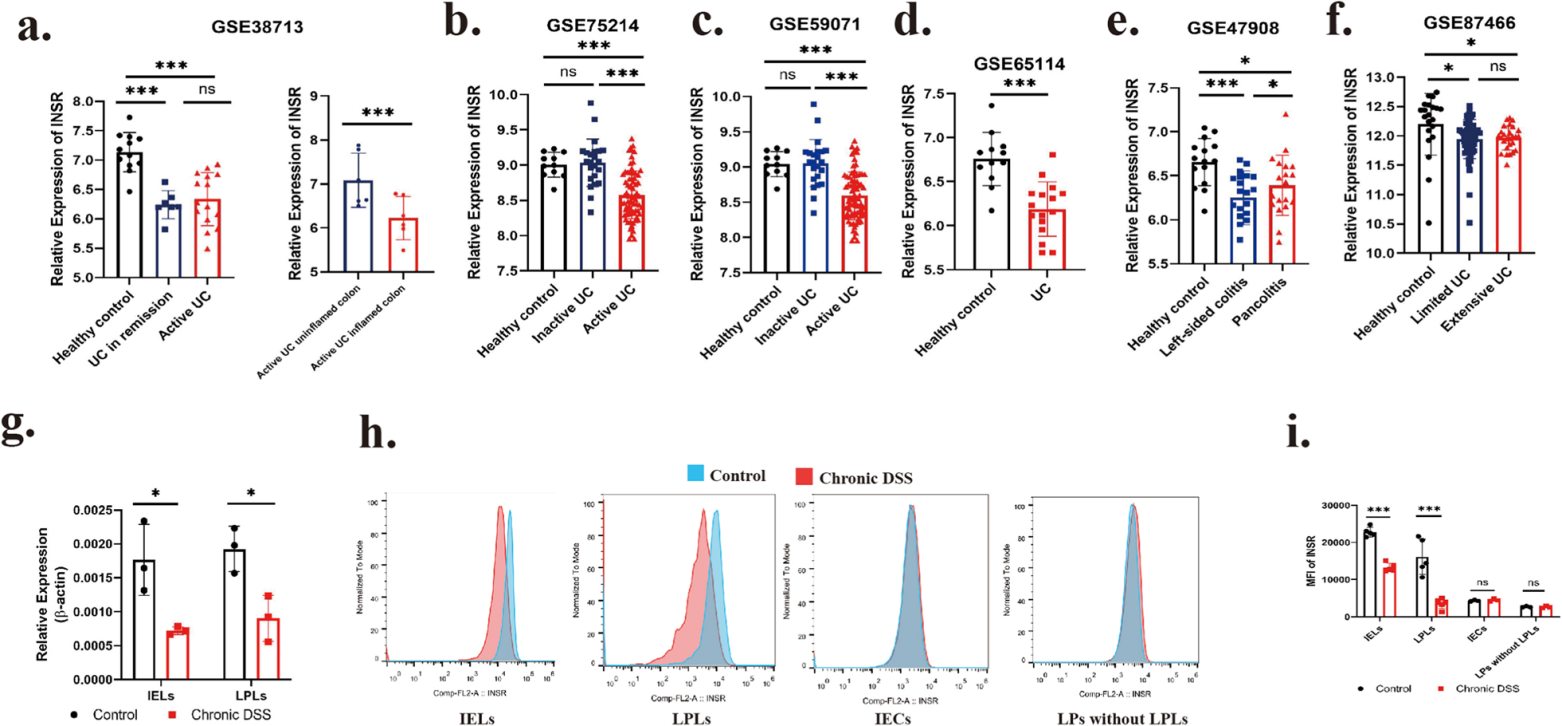
Expression of insulin receptors was significantly reduced in colitis both in IBD patients or animal model. **a**–**f** Expression of INSR in human ulcerative colitis mucosa. Sequencing data GSE38713, GSE75214, GSE59071, GSE65114, GSE47908 and GSE87466 were selected from GEO database. **g** mRNA INSR expression of IELs and LPLs in Control and DSS group were analyzed by qPCR. n = 3. **h**, **i** INSR expression of in IELs, LPLs, intestinal epithelial cells (IECs) and LPs (without LPLs). INSR expression were detected by Flow staining and calculate by Mean fluorescence intensity. (IELs and LPLs: n = 5; IECs and LP cells without LPLs: n = 3)

Since insulin receptor (INSR) expression was significantly reduced in IELs in DSS-induced colitis, INSR expression was analyzed in 6 human sequencing data of UC mucosa from the GEO database to verify whether this trend is equally present in human (Fig. 2a–f). Consistent with results in animal model, it was found that INSR expression was significantly lower in patients with active UC compared to healthy controls in all 6 databases. In particular, INSR expression was also significantly lower in inflamed sites than non-inflamed sites from same UC patient (Fig. 2a). However, since these human sequencing results were obtained from colon tissue rather than single cells, it remains unknown whether the decrease in INSR is universal in colon or limited to specific cells.

To further explore the cellular specificity of down-regulated INSR, the expressions of INSR in lymphocytes from different layer of colon mucosa (IELs from epithelial layer and lamina propria lymphocytes (LPLs) from lamina propria) were analyzed by qPCR. The expression of INSR was found to be down-regulated in both IELs and LPLs in DSS-induced chronic colitis (Fig. 2g). Finally, we examined the protein expression of INSR in different layers and cell types by INSR and CD45 flow staining. In isolated cells from epithelial layer, expression of CD45^+^ represents IELs and CD45- represents epithelial cells. As in isolated cells from lamina propria layer, expression of CD45^+^ represents LPLs and CD45- represents LP cells without lymphocytes. In agreement with the qPCR results, INSR expression was found to be down-regulated in both IELs and LPLs in chronic colitis. However, epithelial cells and lamina propria cells without lymphocytes showed no significant difference in the expression of INSR in chronic colitis (Fig. 2h, i). In conclusion, INSR was specifically down-regulated in lymphocytes in chronic colitis, whereas other colonic cells showed no significant change.

### Stimulation of endogenous insulin secretion by different ways of glucose intake shows distinct effects on DSS-induced chronic colitis

To investigate the roles of insulin pathways on intestinal inflammation, the effects of endogenous insulin in intestinal inflammation were analyzed firstly. Endogenous insulin secretion is positively correlated with blood glucose levels. High-sugar diets stimulate insulin secretion, thereby increasing endogenous insulin acquisition in the colon. Previous studies reported that a high-sugar diet aggravated colitis through intestinal microbiota [43].

An animal model of high-sugar drinking water combined with DSS-induced chronic colitis was established. Three groups of animal models (Control, DSS and DSS + high-glucose) were established in Additional file 1: Figure S4a. High sugar drinking was found to significantly exacerbate DSS-induced chronic colitis, as represented by higher mortality (all mice dead after the 2nd cycle of DSS), high Disease activity index(DAI) scores and shorter colonic length(Additional file 1: Figure S4b–e). An increased mRNA expression of IL-6 and TNF-α was also detected by qPCR (Additional file 1: Figure S4f). However, animal model with high-sugar drinking water inevitably alters the intestinal microbiota, which could not demonstrate the precise effect of the insulin pathway on intestinal inflammation.

An intraperitoneal high glucose (IPHG) was administered to increase endogenous insulin secretion without altering the intestinal microbiota. Three groups of animal models (Control, DSS and DSS + IPHG) were established as Additional file 1: Figure S4g. It was found that intraperitoneal high glucose solution injection failed to affect the severity of DSS-induced colitis, as represented by no significant difference in weight, DAI scores, colonic length, expression of IL-6 and TNF-α and HE staining (Additional file 1: Figure S4h–l). However, prolonged intraperitoneal administration of glucose resulted in ascites in mice. As insulin receptors are widely distributed throughout the body, the tolerated dose of intraperitoneal glucose may not be sufficient to trigger enough insulin secretion to alter intestinal inflammation.

### Activation of insulin receptor by rectal insulin instillation exacerbates DSS-induced chronic colitis

Since the intestinal effects of endogenous insulin were limited due to the distribution of insulin receptors throughout the body, regional administration improves the specificity of insulin. Notably, insulin is unable to enter the circulation system through the intestinal barrier. Therefore, rectal insulin instillation can avoid the systemic effects of insulin and target on colonic mucosal specifically. Mohammad Yassin et al. reported a protective effect of rectal insulin instillation in colon tumor genesis, but its role in colitis remains to be further studied [41]. Therefore, rectal insulin instillation was used to specifically activate the colonic mucosal insulin receptor to investigate the effect of INSR on the intestinal mucosa, especially on mucosal immunity.

**Fig. 3:**
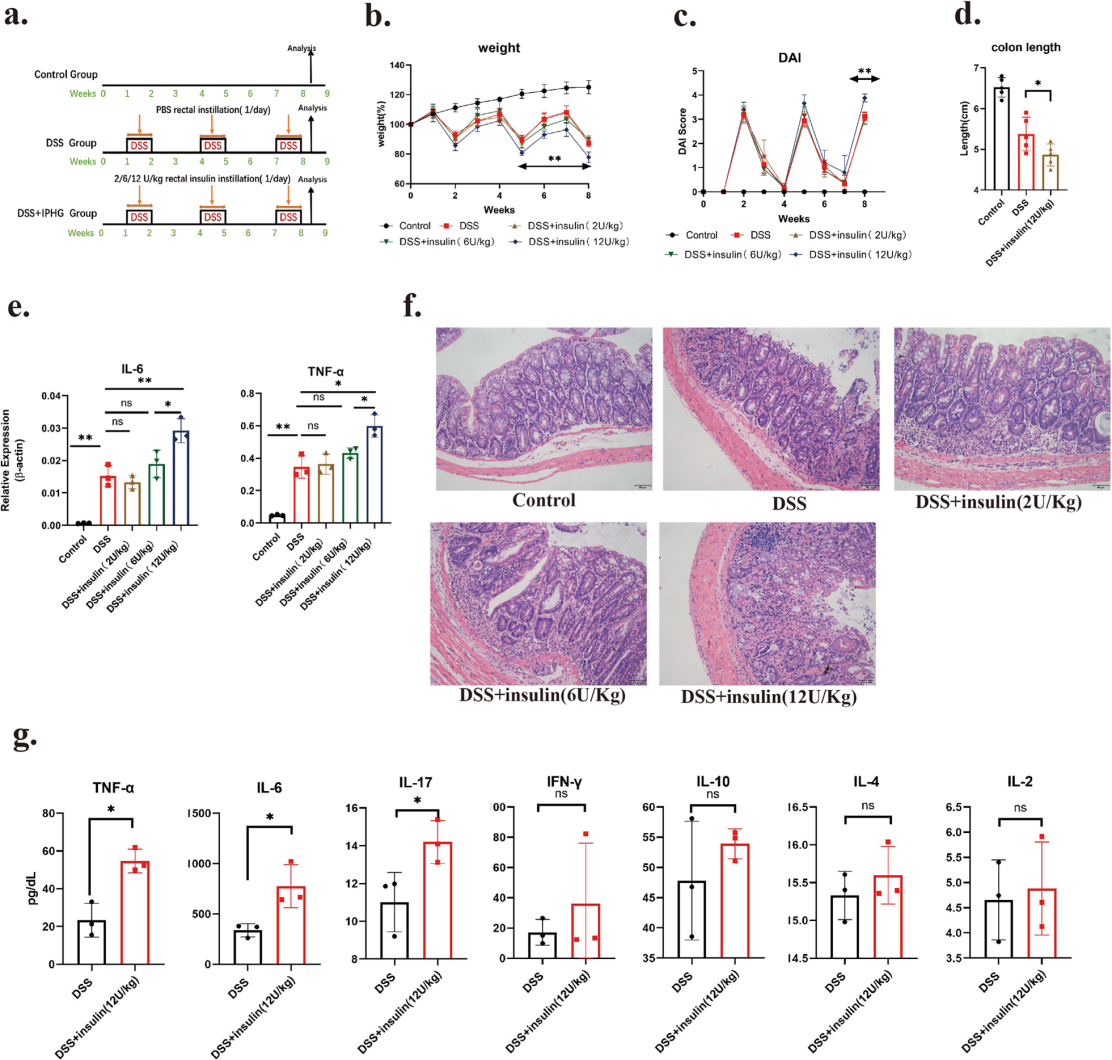
Activation of insulin receptor by rectal insulin instillation exacerbates DSS induced chronic colitis. A animal model schematic outline of Control group, DSS group and DSS + insulin group. 2U, 6U, 12U/Kg insulin were used in different DSS + insulin groups. Five mice at least per group were analyzed. **b**–**d** Weight change, DAI score and colon length of Control group, DSS group and DSS + insulin group. n ≥ 5. **e** mRNA expression of IL-6 and TNF-ɑ in Control group, DSS group and DSS + insulin group were analyzed by qPCR. n ≥ 3. **f** representative histological sections of the colon from Control group, DSS group and DSS + insluin group. Sections were stained with hematoxylin and eosin. **g** Concentration of TNF-ɑ, IL-6, IL-17, IFN-γ, IL-10, IL-4 and IL-2 in colon segment culture supernatant were analyzed by Cytometric Bead Array(CBA). n = 3

Three groups of animal models (Control, DSS and DSS + insulin) were established in Fig. 3a. The severity of the colitis was evaluated by body weight, colon length, DAI score, HE staining and expression of TNF-α and IL-6. Compared with the DSS group, it was observed that mucosal infiltrating lymphocytes and tissue inflammatory cytokines were increasing with insulin concentration in DSS + insulin group (Fig. 3e, f). DSS + insulin(12U/Kg) group showed the most down-regulated body weight and colonic length, an up-regulated DAI score as well as expression of TNF-α and IL-6 (Fig. 3b–e). The most severe intestinal villin destruction and mucosal inflammatory infiltration also occurred in the DSS + insulin(12U/Kg) group. These results suggest that rectal insulin instillation could aggravate DSS-induced chronic colitis.

To investigate the underlying immunological mechanisms of colitis aggravated by rectal insulin instillation, inflammatory factors in the colon were detected by full-thickness colonic tissue culture and Cytometric Bead Array [39]. The concentration of TNF-ɑ, IL-6, IL-17, IFN-γ, IL-10, IL-4 and IL-2 in the supernatant of intestinal segment culturing medium was detected by Cytometric bead array (CBA). We noticed that the expression of IL-6, TNF-ɑ and IL-17 was significantly up-regulated in DSS + insulin(12U/Kg) group compared with the DSS group (Fig. 3g). Whereas expression of IFN-γ, IL-2, IL-4 and IL-10 showed no significant difference between two groups. TNF-ɑ and IL-17 are the most important proinflammatory cytokines in IBD.TNF-ɑ targeted drugs has been used clinically (infliximab) [6, 7]. The increased TNF-ɑ and IL-17 contribute to the aggravation of intestinal inflammation directly.

### Rectal insulin instillation leads to disrupted subgroups and promoted inflammatory cytokines secretion in IELs

**Fig. 4:**
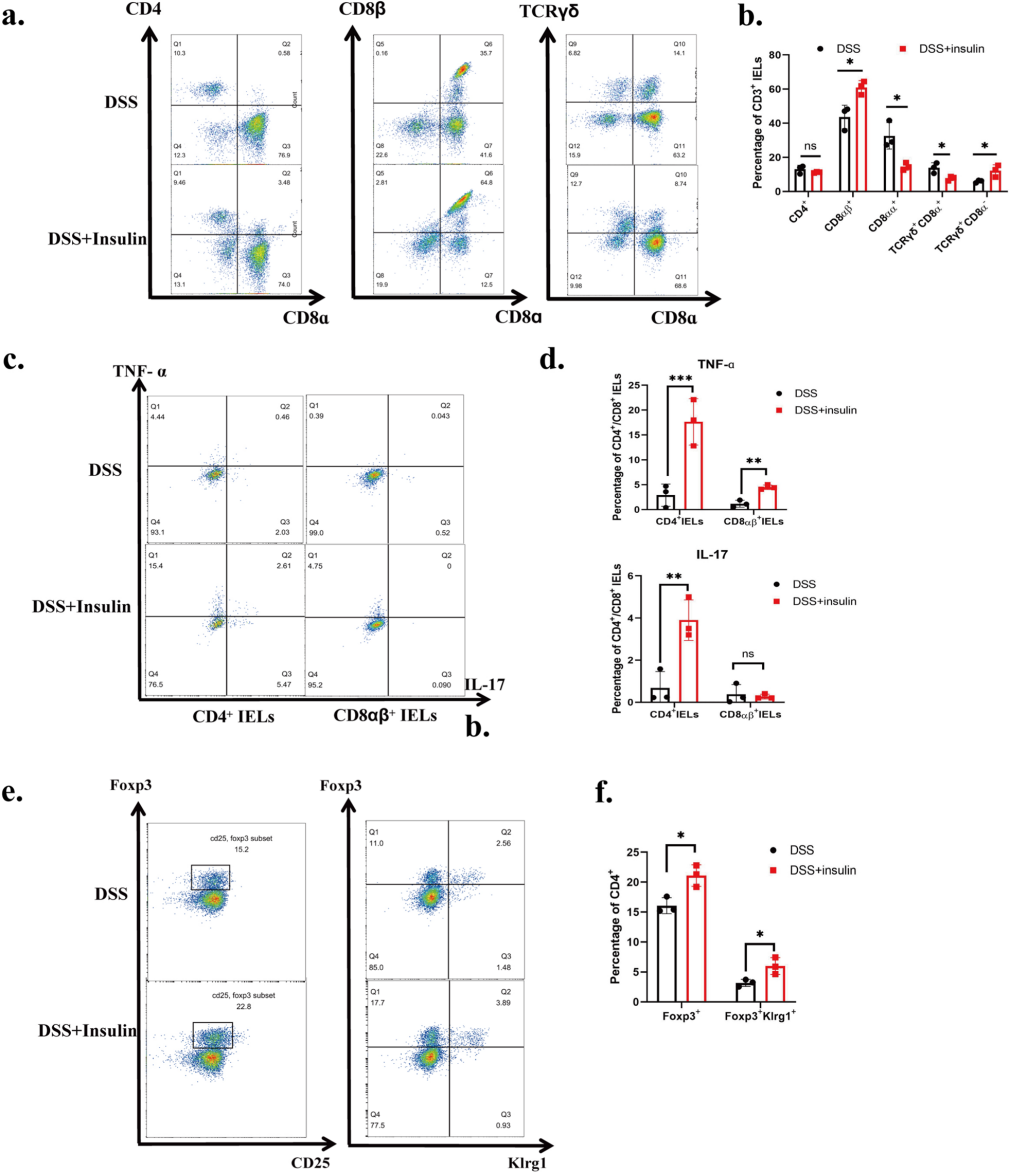
Rectal insulin instillation may exacerbate colitis by disrupting IELs’ subgroup and promoting inflammatory factors. Representative plots of cell-surface staining of CD4, CD8α, CD8β and TCRγδ in colonic IELs from DSS group and DSS + insulin(12U/Kg) group. IELs were gated on CD3 + live cells. Percentages of the indicated makers in colonic IELs of DSS group and DSS + insulin(12U/Kg) group. n = 3. The findings of three pooled independent experiments are shown. Two independent experiments were performed, with similar results, and one experiment were used for statistic. **c** Representative plots of Intracellular-staining of TNF-ɑ, IL-17 in colonic CD4 + and CD8αβ + IELs from DSS group and DSS + insulin(12U/Kg) group.CD4 + and CD8αβ + IELs were gated on CD3 + live cells. **d** Percentages of TNF-ɑ, IL-17 in colonic CD4 + and CD8αβ + IELs of DSS group and DSS + insulin(12U/Kg) group. n = 3. The findings of three pooled independent experiments are shown. **e** Representative plots of foxp3, CD25 and Klrg1 in colonic CD4 + LPLs from DSS group and DSS + insulin(12U/Kg) group. LPLs were gated on CD3 + CD4 + live cells. **f** Percentages of foxp3, Klrg1 in colonic CD4 + LPLs of DSS group and DSS + insulin(12U/Kg) group. n = 3.The findings of three pooled independent experiments are shown

Since biomolecules such as insulin are difficult to pass through the intestinal barrier, IELs, which localize between the intestinal epithelium and sense intestinal luminal molecules [44], are the most possible immune target cells and source of Inflammatory cytokines in rectal insulin instillation. Therefore, we detected the subgroups and the secretion of inflammatory cytokines in IELs. As shown in Fig. 4a, b, an increased percentage of CD8αβ and TCRγδ^+^CD8^−^, an decreased percentage of CD8aa and TCRγδ^+^CD8^+^, and unchanged CD4^+^ and total TCRγδ^+^ were detected. As previously described, CD4^+^ and CD8αβ^+^IELs show a pro-inflammatory effect by secreting inflammatory cytokines represented by TNF-α and IL-17, and CD8αα^+^ IELs show an anti-inflammatory effect [16, 21, 23]. These observed changes in IEL subgroups represent an increase in a pro-inflammatory subgroup and a decrease in an anti-inflammatory subgroup (Fig. 4a, b). Then, the expression of TNF-ɑ and IL-17 in CD4^+^ and CD8αβ^+^ IELs was tested by intracellular staining. Compared with the DSS group, the DSS + insulin group showed more TNF-ɑ secretion in both CD4 and CD8αβ IELs. Besides, IL-17 was not expressed in CD8αβ^+^ IELs, but a significantly up-regulated expression in CD4^+^ IELs was detected (Fig. 4c, d). Notably, the percentage of CD4^+^ IEL remained unchanged while its inflammatory factor secretion was significantly up-regulated. Taken together, these results indicate that disrupted subgroups and increased TNF-ɑ and IL-17 in IELs contribute to exacerbated colitis.

Since Treg resides in lamina propria and MLNs are pivotal for intestinal immunity and IBD [8], flow staining of lamina propria and mesenteric lymph node CD3^+^CD4^+^Foxp3^+^ Treg cells was operated. An increase of Treg in the DSS + insulin group was detected (Fig. 4e, f). By comparison of lamina propria Treg with mesenteric lymph node Treg, it was found that intestinal lamina propria Treg express klrg1 instead of classic marker CD25. In addition, Klrg1^+^ Treg was significantly upregulated after rectal insulin instillation (Fig. 4e, f). Available studies suggest that klrg1^+^Treg is more profoundly activated and more immunosuppressive than klrg1^−^Treg [45, 46]. The mechanisms of the altered Treg phenotype require further study.

Insulin is unable to cross the intestinal barrier and theoretically does not act on secondary lymphoid tissue. To investigate whether rectal insulin instillation affected the mesenteric lymph nodes, we examined changes in TNF-ɑ, IL-17 secreting cells and Treg in MLN. No significant changes in the MLN were found following rectal insulin instillation (Additional file 1: Figure S5), suggesting that rectal insulin instillation targets the colonic mucosa specifically. In conclusion, rectal insulin instillation increased the proportion of pro-inflammatory IEL subpopulations as well as inflammatory factor secretion, and decreased the proportion of the anti-inflammatory IELs, leading to the exacerbation of colitis.

### Rectal insulin instillation enhances EZH2 expression and EZH2 inhibitor GSK126 reverses colitis exacerbation induced by rectal insulin instillation

We expected to use INSR-specific inhibitors and further investigate their effects on colitis. However, there is no specific INSR inhibitor at present due to the similarity between INSR and insulin-like growth factor receptor (IGF-R) [47]. GSK1904529A is a co-inhibitor of INSR and IGF-R [48]. We attempted to use GSK1904529A by rectal instillation and detect the severity of colitis. However, by comparing the DSS group and DSS + GSK1904529A group in terms of colon length, body weight and DAI, it was found that GSK1904529A rectal instillation did not reduce colitis but rather aggravated it (Additional file 1: Figure S5).

To explore INSR and insulin pathway-based drugs for IBD, the mechanism underlying disrupted IELs’ subgroups and promoted inflammatory cytokines were further investigated. As previously described, glucose metabolism leads to epigenetic alterations in T cells [24, 28].EZH2 is a key factor in regulation of H3K27 methylation modifications, one of major pattern of epigenetic modification [33, 49, 50]. Here, we show that EZH2 contribute to the disruption of IEL subgroups and the promotion of inflammatory factors induced by rectal insulin instillation.

**Fig. 5:**
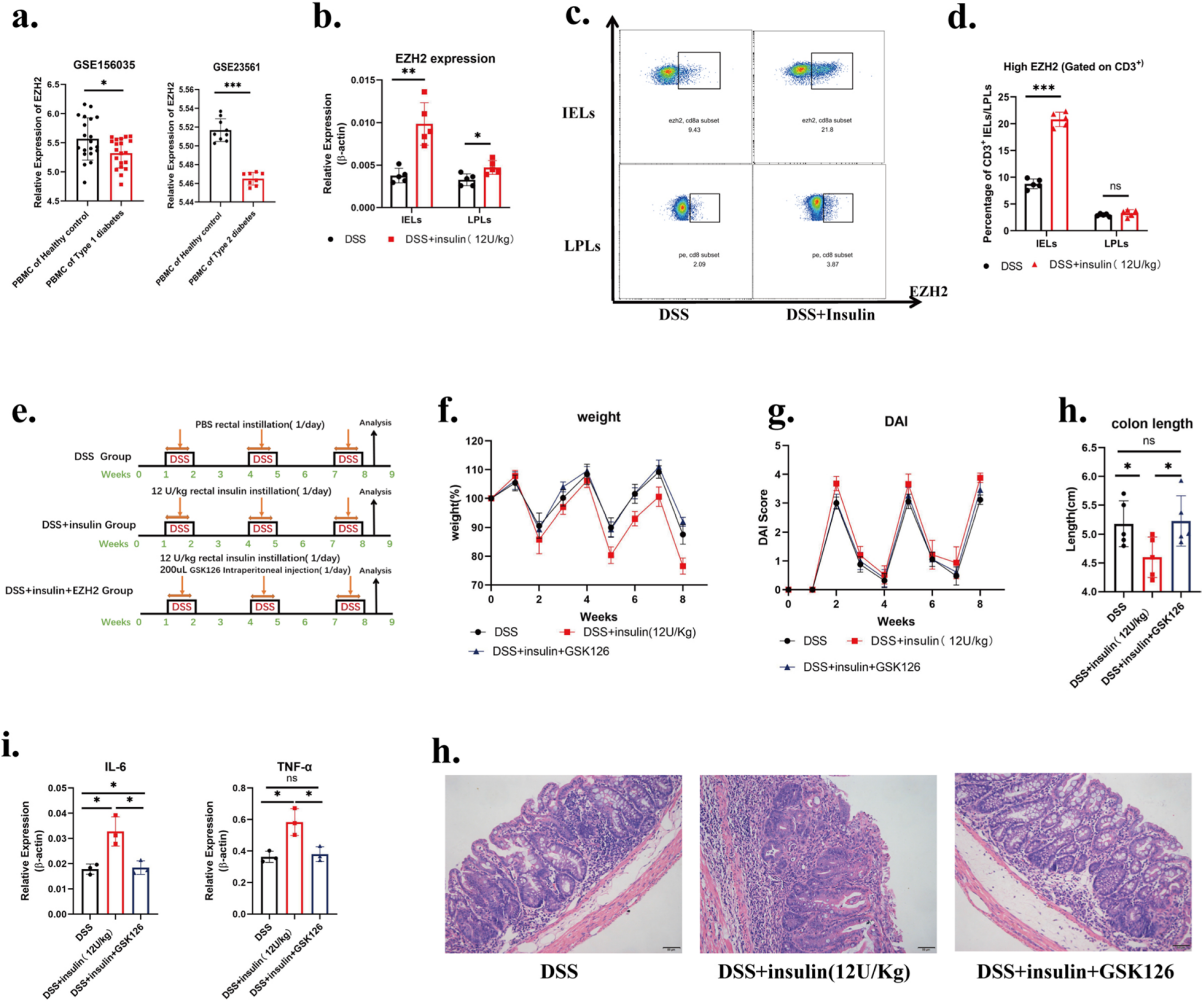
Rectal insulin instillation enhances EZH2 expression and EZH2 inhibitor GSK126 reverses colitis exacerbation induced by rectal insulin instillation. **a** Differential Expression of EZH2 in human PBMC from T1D and T2D patients. Sequencing data GSE156035,GSE23561 were selected from GEO database. **b** mRNA expression of EZH2 in DSS group and DSS + insulin group were analyzed by qPCR. n ≥ 5. **c** Representative plots of Intracellular-staining of EZH2 in colonic IELs and LPLs from DSS group and DSS + insulin(12U/Kg) group.IELs and LPLs were gated on CD3 + live cells. **d** Percentages of high-EZH2 cells in colonic IELs and LPLs of DSS group and DSS + insulin(12U/Kg) group. n = 5. **e** A animal model schematic outline of Control group, DSS + insulin(12U/Kg) group and DSS + insulin(12U/Kg) + GSK126 group. Five mice at least per group were analyzed. **f**–**h** weight change, DAI score and colon length of Control group, DSS + insulin(12U/Kg) group and DSS + insulin(12U/Kg) + GSK126 group. n ≥ 5. **i** mRNA expression of IL-6 and TNF-ɑ in Control group, DSS + insulin(12U/Kg) group and DSS + insulin(12U/Kg) + GSK126 group were analyzed by qPCR. n = 3. **j** representative histological sections of the colon from Control group, DSS + insulin(12U/Kg) group and DSS + insulin(12U/Kg) + GSK126 group. Sections were stained with hematoxylin and eosin

**Fig. 6:**
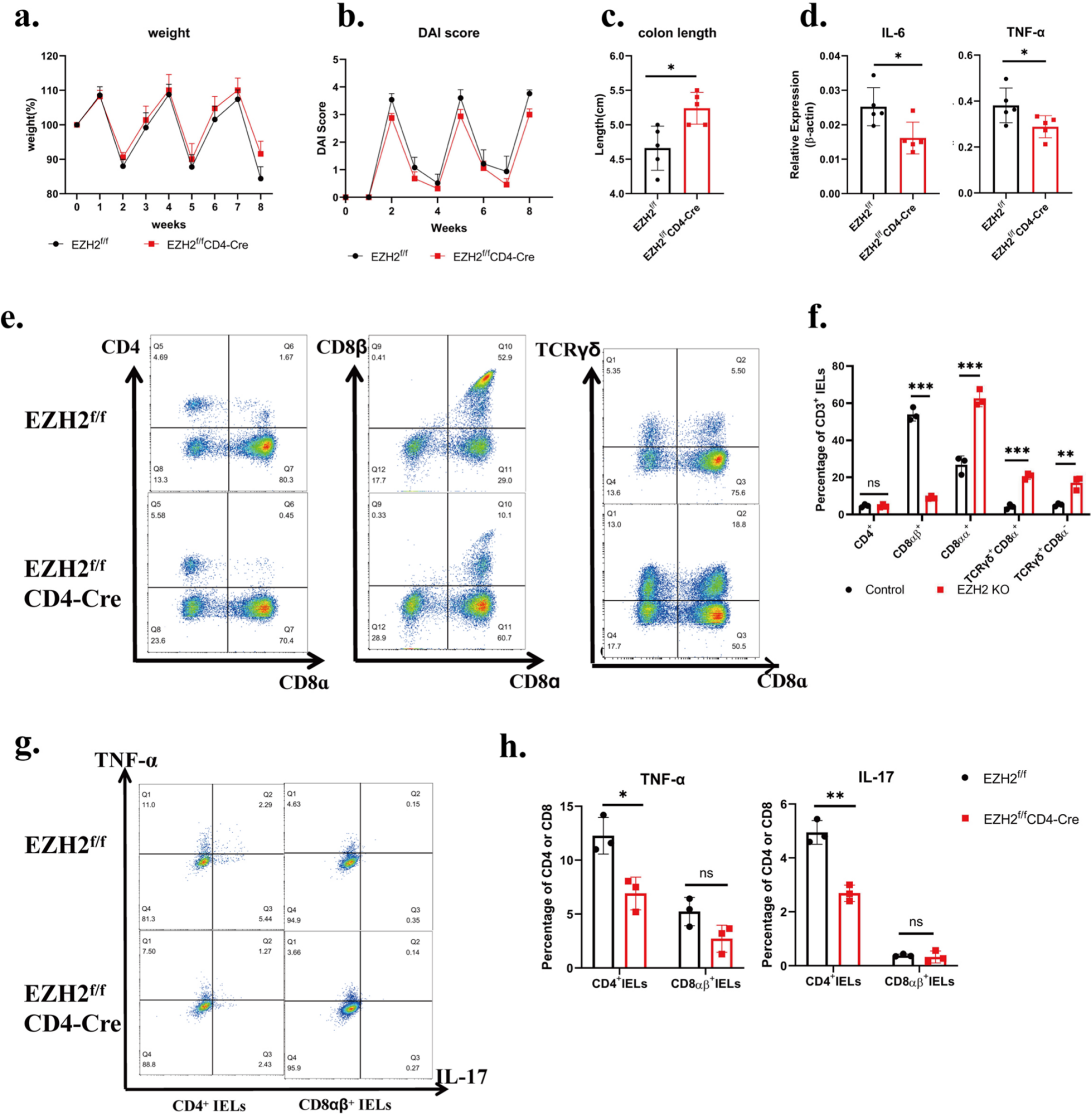
EZH2 knockout alleviates colitis and exhibits opposite IELs’ phenotype to rectal insulin instillation. **a**–**c**. weight change, DAI score and colon length of EZH2^fl/fl^CD4^cre^ mice and EZH2^fl/fl^ mice in DSS induced chronic colitis. n ≥ 5. d.mRNA expression of IL-6 and TNF-ɑ of EZH2^fl/fl^CD4^cre^ mice and EZH2^fl/fl^ mice in DSS induced chronic colitis were analyzed by qPCR. n ≥ 5. **e** Representative plots of cell-surface staining of CD4,CD8α,CD8β and TCRγδof colonic IELs in DSS induced chronic colitis from EZH2^fl/fl^CD4^cre^ mice and EZH2^fl/fl^ mice. IELs were gated on CD3 + live cells. **f** Percentages of the indicated makers of colonic IELs in DSS induced chronic colitis from EZH2^fl/fl^CD4^cre^ mice and EZH2^fl/fl^ mice. n ≥ 3. The findings of three pooled independent experiments are shown. Two independent experiments were performed, with similar results, and one experiment were used for statistic. **g** Representative plots of Intracellular-staining of TNF-ɑ, IL-17 of colonic CD4 + and CD8αβ + IELs in DSS induced chronic colitis from EZH2^fl/fl^CD4^cre^ mice and EZH2^fl/fl^ mice.CD4 + and CD8αβ + IELs were gated on CD3 + live cells. Three independent experiments were performed, with similar results,and one experiment were used for statistic. **h** Percentages of TNF-ɑ, IL-17 in colonic CD4 + and CD8αβ + IELs in DSS induced chronic colitis from EZH2^fl/fl^CD4^cre^ mice and EZH2^fl/fl^ mice. n = 3

The insulin pathway is inhibited in type 1 and type 2 diabetes. By analyzing sequencing data of type 1 and type 2 diabetes patients from GEO Databases, EZH2 expression was found to be reduced in PBMC of both type 1 diabetes and type 2 diabetes patients (Fig. 5a). We further examined whether rectal insulin instillation caused alterations in EZH2 expression. It was detected that the expression of EZH2 mRNA in IELs was significantly upregulated after rectal insulin instillation. LPLs exhibited the same tendency but to a lesser extent (Fig. 6b). Furthermore, the expression of EZH2 in the protein level was examined by Flow staining. A significant upregulation in IELs was detected, but no significant difference in LPLs (Fig. 5c, d). The difference in EZH2 expression alternation between IELs and LPLs is resulted from the different mucosal localization. Rectal insulin instillation act directly on the epithelium, whereas it can only act on the lamina propria at the site with a broken epithelium barrier [51].

To investigate whether EZH2 is involved in the exacerbation of colitis by INSR, the role of EZH2 in colitis was examined by using the EZH2-specific inhibitor GSK126 [52]. The experiment was constructed as shown in Fig. 5e. GSK126 was injected intraperitoneally and the severity of colitis was measured as mentioned before. EZH2 intervention could alleviate the exacerbation of colitis caused by rectal insulin installations, as evidenced by less weight loss, less DAI score, fewer inflammatory cytokines, and a longer colon length (Fig. 5f–h). These results suggest that rectal insulin instillation promotes EZH2 expression in intestinal mucosal T cells and inhibition of EZH2 could reverse exacerbated colitis induced by rectal insulin instillation.

### EZH2 knockout alleviates colitis and exhibits the opposite phenotype to rectal insulin instillation

To investigate the effect and mechanism of EZH2 on regulating IELs and colitis, T cell EZH2-specific knockout mice (EZH2^fl/fl^CD4^cre^) were constructed by crossing EZH2^fl/fl^ mice with CD4^cre^ mice. Since both CD4 and CD8 T cells experienced thymic agonist selection, EZH2 was eliminate in both CD4^+^ and CD8^+^ T cells. In DSS-induced chronic colitis, EZH2^fl/fl^CD4^cre^ mice showed less severity of colitis than control mice (EZH2^fl/fl^), exhibiting less weight loss, DAI score, inflammatory cytokines, and longer colon length (Fig. 6a–d). Furthermore, flow-staining revealed a significantly decreased CD8αβ IELs and an increased CD8aaTCRγδ IELs, which is opposite to the IEL phenotype in rectal insulin instillation (Fig. 6e, f). A decrease in TNF-α and IL-17 secreting CD4 IELs was also detected, while there was no significant difference in TNF-α or IL-17 secreting CD8αβ IELs (Fig. 6g, h). The alternations of IELs subgroups and inflammatory cytokines showed in EZH2 knockdown mice are opposite to those in rectal insulin instillation, suggesting that EZH2 may act as a downstream molecule of insulin pathway. These results identified increased EZH2 expression contributes to the alternations of IELs’ subgroups and inflammatory cytokines by rectal insulin instillation.

### Disrupted IELs’ phenotype and increased inflammatory cytokines by rectal insulin instillation originated from upregulated colonic tissue resident memory T cells

**Fig. 7:**
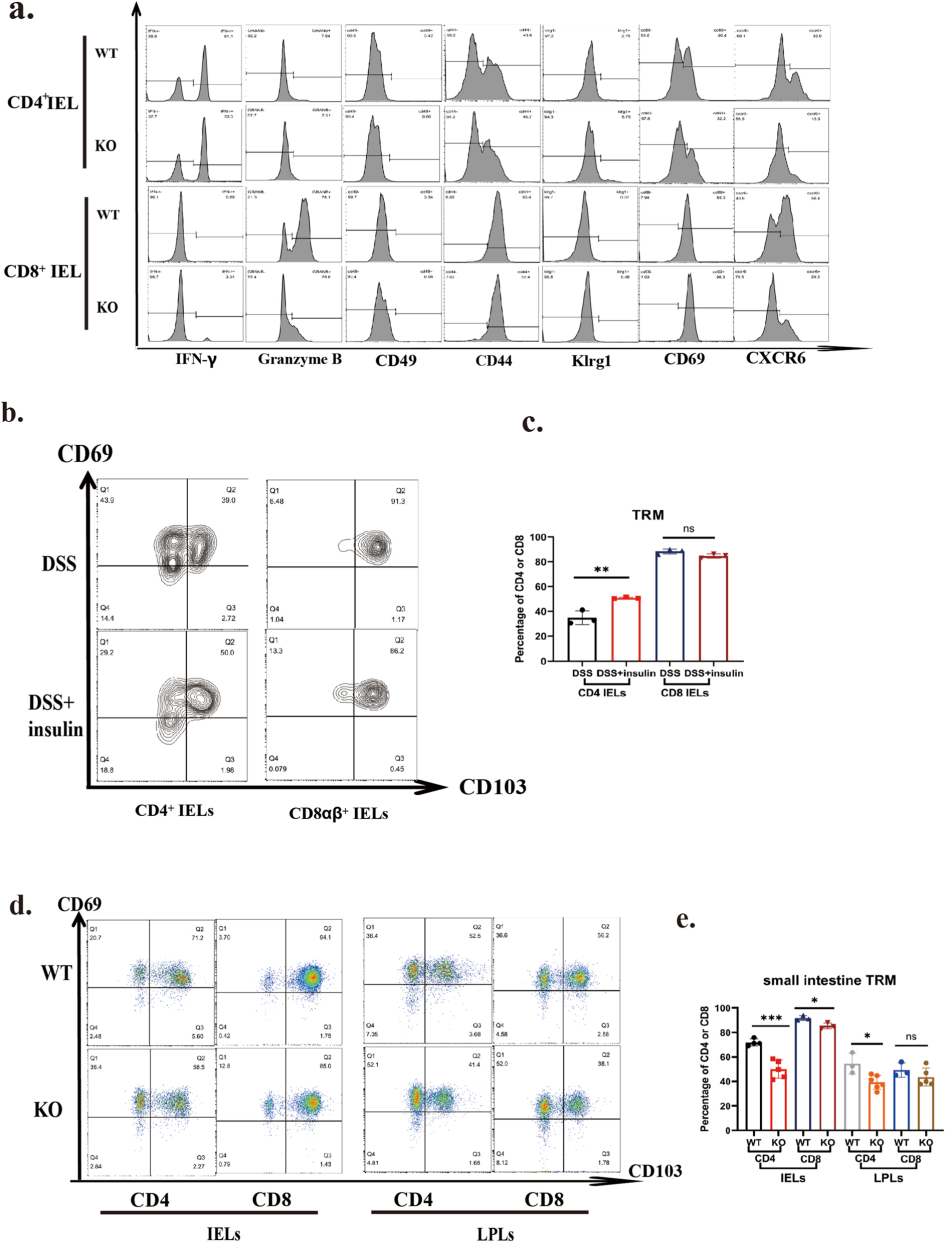
Disrupted IELs’ Phenotype and increased inflammatory factors by rectal insulin instillation originated from upregulated colonic TRM. **a** Representative plots of staining of IFN-γ, Granzyme B, CD49, CD44, Klrg1, CD69 and CXCR6 of colonic IELs from KO(EZH2^fl/fl^CD4^cre^)mice and WT(EZH2^fl/fl^)mice. n ≥ 3. IELs were gated on CD3 + live cells. **b** Representative plots of cell-surface staining of CD69, CD103 of colonic IELs in DSS group and DSS + insulin(12U/Kg) group. IELs were gated on CD3 + live cells. **c** Percentages of the CD69 + CD103 + cells of colonic IELs in DSS group and DSS + insulin (12U/Kg) group. Two independent experiments were performed with similar results (n ≥ 3), and one experiment were used for statistic. **d** Representative plots of cell-surface staining of CD69,CD103 of small intestine IELs from EZH2^fl/fl^CD4^cre^ mice and EZH2^fl/fl^ mice. IELs were gated on CD3 + live cells. **e** Percentages of the CD69 + CD103 + cells of small intestine IELs from EZH2^fl/fl^CD4^cre^ mice and EZH2^fl/fl^ mice. Two independent experiments were performed (n ≥ 3), with similar results, and one experiment were used for statistic

To further investigate the mechanism of EZH2 in regulating the subgroups of IELs and inflammatory cytokines, functional marker of T cells [16] including IFN-γ, Granzyme B, CD44, CD49, Klrg1, CD69, CXCR6 were analyzed in CD4^+^IELs and CD8^+^IELs from EZH2^fl/fl^(WT) and EZH2^fl/fl^CD4^cre^ (KO)mice. As shown in Fig. 7a, CXCR6, Granzyme B expression was significantly decreased in CD4^+^and CD8^+^IELs from KO mice.CD69 expression was significantly decreased in CD4^+^IELs from KO mice, but not in CD8^+^IELs.No significant differences were detected in the expression of IFN-γ, CD44, CD49 and Klrg1 between KO and WT mice.

Current studies have shown that both CXCR6 and CD69 are highly expressed in intestinal resident memory T cells (TRM), which is crucial for the induction of IBD-related chronic inflammation [12]. CXCR6 is one of the representative markers of TRM, which is closely related to T cell colonization, survival and proliferation [53]. Expression of CXCR6 represents T cell colonization and long-term presence in colonic mucosa. CD69, a crucial maker for T-cell activation, is the most important marker of TRM. Highly expressed CD69 in TRM represents its activated state including secretion of inflammatory cytokines and cytotoxic effects [11]. Multiple studies have shown the powerful capacity of TRM in secreting TNF-α and IL-17, especially CD4^+^TRM [15, 54, 55]. In the current study, it was also found that most intestinal mucosal TNF-α or IL-17-secreting T cells highly express CD69 (Additional file 1: Figure S7).

The alternations of TRM-related phenotype suggest the disrupted IELs’ subgroups and increased inflammatory cytokines after rectal insulin instillation may result from the increased TRM promoted by EZH2. During ongoing intestinal inflammation, Naïve T cells activate into effector T cells and migrate to the intestine. Effector T cells could further differentiate into TRM [56]. The increased TRM colonized in intestinal epithelial layer or lamina propria layer, which turned into IELs or LPLs respectively. Increased TRM in IELs or LPLs altered the subpopulation of IELs (increased pro-inflammatory) and up-regulated the intestinal mucosal inflammatory factors (IL-17, TNF), which ultimately led to exacerbation of intestinal inflammation.

To investigate the role of rectal insulin instillation in the regulation of TRM, the alterations of colonic TRM within IELs after rectal insulin instillation were examined. As expected, rectal insulin instillation was found to induce a significant increase of CD4^+^ TRM, while no significant difference was found in CD8^+^ IELs (Fig. 7b, c).

Furthermore, to investigate the role of EZH2 in the regulation of TRM, the alterations of TRM in the epithelial and lamina propria of the small intestine from KO and WT mice were tested. T cell-specific knockdown of EZH2 resulted in a significant reduction of CD4^+^ TRM in both the epithelial and lamina propria layers of the small intestine. However, CD8^+^ TRM showed a relatively small reduction in the epithelial layer of the small intestine (Fig. 7d, e). These results suggest that EZH2 expression contributes to the differentiation of CD4^+^ and CD8^+^TRM in small intestine, especially for CD4^+^ TRM. In conclusion, these results identified rectal insulin instillation could promote TRM differentiation through EZH2, thus leading to the disrupted IELs’ subgroups and increased inflammatory cytokines.

### Requirement for EZH2 expression in the differentiation of colonic mucosal CD4 and CD8 tissue resident memory T cells

**Fig. 8:**
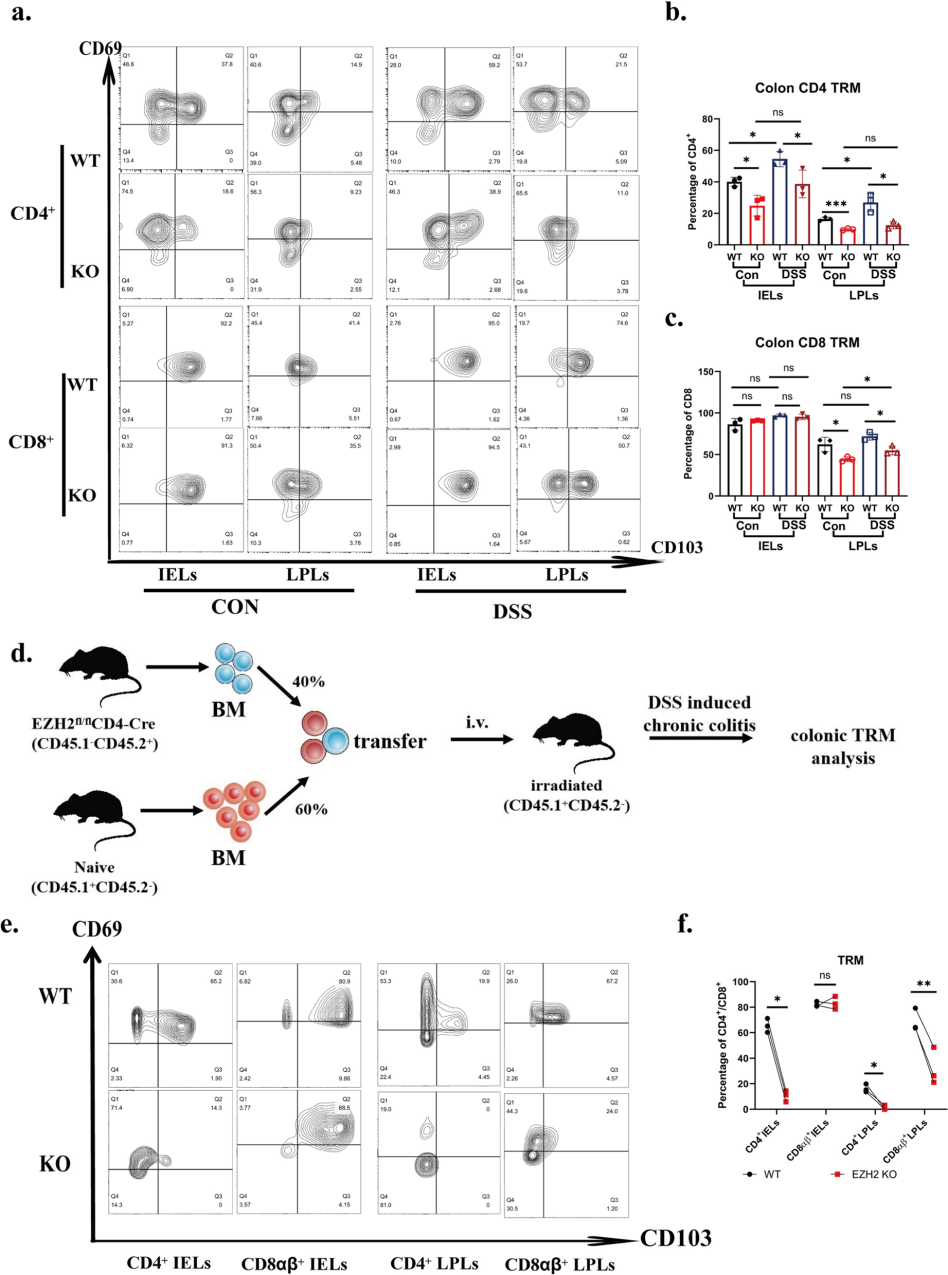
Requirement for EZH2 expression in differentiation of colonic mucosal CD4 and CD8 tissue resident memory T cells. Representative plots of cell-surface staining of CD69, CD103 of colonic IELs and LPLs from EZH2^fl/fl^CD4^cre^ mice and EZH2^fl/fl^ mice with or without DSS induced chronic colitis. IELs were gated on CD3 + live cells. **b**, **c** Percentages of the CD69 + CD103 + cells of colonic IELs from EZH2^fl/fl^CD4^cre^ mice and EZH2^fl/fl^ mice with or without DSS induced chronic colitis. Two independent experiments were performed (n ≥ 3), with similar results, and one experiment were used for statistic. **d** A animal model of BM chimeras. A BM chimeras were generated by mixing congenitally marked BM cells from Ezh2fl/flCD4-Cre mice (EZH2 knockdown; CD45.2, 40%) with BM cells from WT mice (CD45.1, 60%).Three mice at least per group were analyzed. **e** Representative plots of cell-surface staining of CD69,CD103 of colonic IELs and LPLs from BM chimeras mice in induced chronic colitis. IELs were gated on CD3 + live cells. **f** Percentages of the CD69 + CD103 + cells of colonic IELs and LPLs from BM chimeras mice in induced chronic colitis. n = 3

To investigate the role of EZH2 in regulating TRM and colitis, colonic epithelium and lamina propria CD4^+^ and CD8^+^ TRM in chronic DSS-induced colitis were detected by flow staining in EZH2^fl/fl^ (WT) and EZH2^fl/fl^CD4^cre^(KO)mice. By comparing control(CON) and chronic colitis(DSS) group in WT mice, CD4^+^TRM was found to be significantly increased in either the epithelium or lamina propria in chronic colitis, whereas CD8^+^ TRM did not show significant alterations in chronic colitis, suggesting that CD4^+^TRM exhibits a more prominent role for in chronic colitis. Conversely, in EZH2-KO mice, colonic epithelial and lamina propria CD4^+^ TRM did not show significant upregulation in DSS group compared with CON group, suggesting that EZH2 knockdown inhibited the upregulation of CD4^+^TRM in chronic colitis (Fig. 8a–c).

By comparing TRM in WT and KO mice, it was found that EZH2 knockdown resulted in a significant downregulation of CD4^+^TRM in both IELs and LPLs, whether in CON or DSS group. EZH2 knockdown similarly resulted in a significant decrease in CD8^+^ TRM in the colonic lamina propria in CON or DSS group. However, neither chronic inflammation nor EZH2 knockdown leads to significant alterations in CD8^+^ TRM in the intestinal epithelium layer (Fig. 8c). These results suggest that increased EZH2 expression contributes to TRM differentiation, especially for CD4^+^TRM.

To evaluate the role of cell-autonomous EZH2 more precisely in the mechanism regulating endogenous TRM differentiation, we generated BM chimeras by mixing congenitally marked BM cells from KO mice (CD45.2, 40%) with BM cells from WT mice (CD45.1, 60%) (Fig. 8d). After 9 weeks of DSS-induced chronic colitis, a decreased percentage of TRM in CD4^+^IELs, CD4^+^LPLs and CD8^+^LPLs were observed among T cells originating from Ezh2fl/fl ERT2-Cre mice compared with that of cells of WT origin. However, there is still no significant difference in CD8^+^IELs (Fig. 8e, f). Altogether, these data highlighted the importance of cell-intrinsic EZH2 in the regulation of TRM differentiation in intestinal inflammation.

## Discussion

**Fig. 9:**
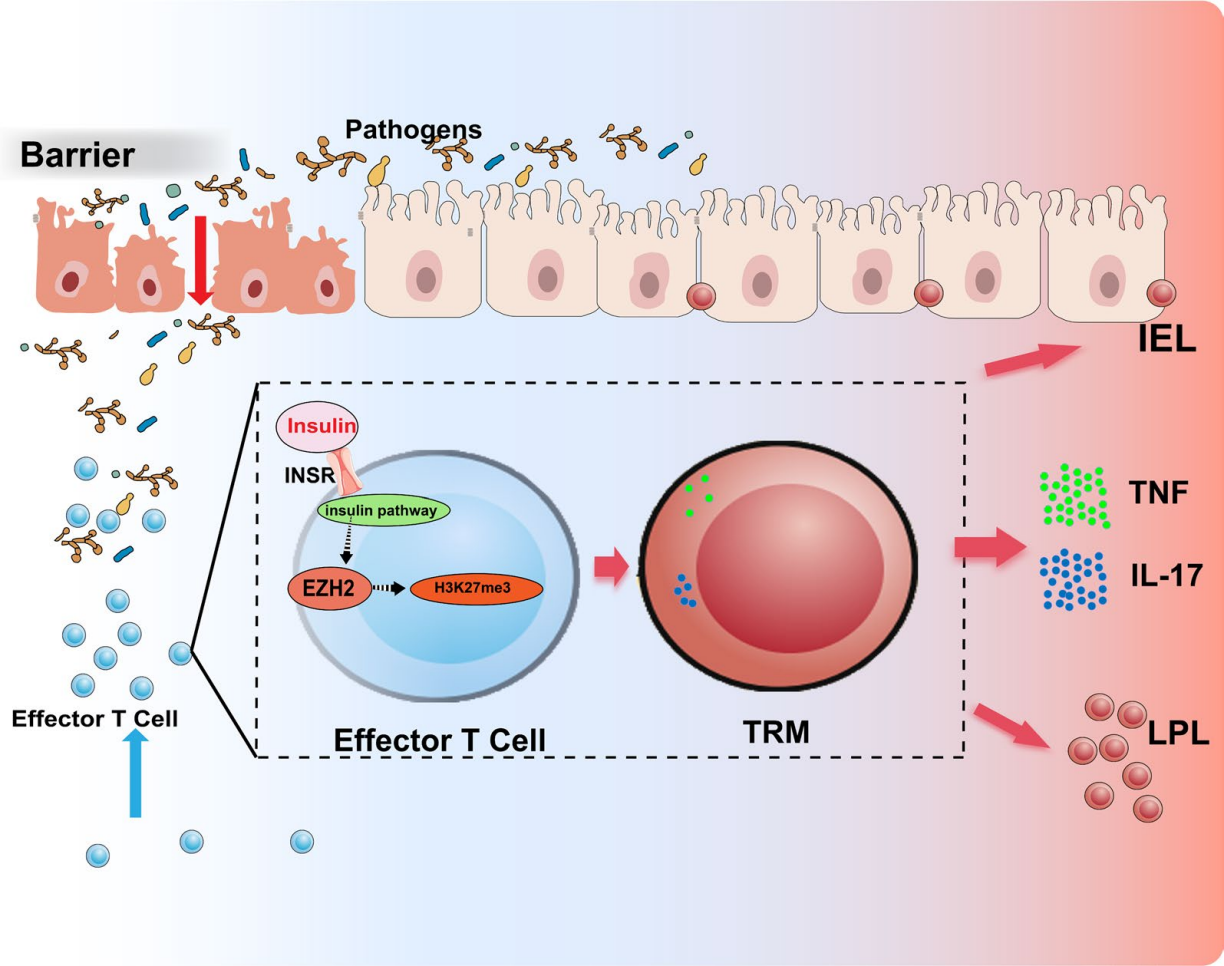
Insulin receptor of intestinal T-cells promote intestinal TRM differentiation via EZH2 and consequently exacerbate intestinal inflammation. In response to pathogen invasion following barrier damage, large numbers of effector T cells proliferate and localise in the gut. Insulin acting on the insulin receptor and activating the insulin pathway, promoting up-regulation of EZH2 by effector T cells. EZH2 promotes the differentiation of effector T cells into TRM through methylation of the H3K27 on specific genes locus.TRM can be permanently retained in the intestinal interepithelial or lamina propria and continuously secrete inflammatory factors such as TNF and IL-17, which ultimately promote intestinal inflammation and lead to the pathogenesis and development of IBD

Metabolism profoundly regulates the function of immune cells [24, 57, 58]. Metabolic therapy had achieved remarkable success in enhancing the efficacy of immunooncological therapies such as PD-1, CART and oncolytic virus [24, 59, 60]. However, different subsets of differentiated T cells utilize distinct metabolic programmes [61, 62]. Conventional naive T cells exist in a resting state with minimal metabolic activity; activated T cells (effector T cells) in inflammation or cancer rapidly upregulate glucose uptake, and turn on aerobic glycolysis and tricarboxylic acid (TCA) cycle, which enables nucleotide synthesis for rapid proliferation and ATP production [63, 64]; a certain number of effector T cells differentiate into TRM [11], however, insights into the metabolic mechanism of TRM differentiation are still lacking. Here, we show that insulin, master regulator of glucose metabolism, can regulate the differentiation of TRM in intestinal mucosa through the histone methylation regulator EZH2. This study provides a novel strategy for immunometabolic therapies in inflammation and cancer therapy (fig. 9).

Drug repurposing is defined as the exploration of alternative indications for already approved drugs, which has been also studied in autoimmune disease research [65]. Drug repurposing could greatly reduce the time and funds in drug development. The wide distribution of insulin receptors throughout the body and their potent metabolic regulation suggest that they may play an crucial role in a variety of diseases. On the other hand, insulin has a well-proven clinical safety profile and a robust industrial production system. Thus, insulin fits extremely well with drug repurposing. Based on the systemic effects of the insulin, we believe that use of insulin as repurposing drug should be based on the targeted delivery systems such as nanomaterials, which could act specifically on certain tissues or cells.

RNA-seq analysis was performed to identify the dysregulated RNA profile using the whole colon RNA isolated from the mouse models of colitis or IBD patients. However, the differential expression can be attenuated or eliminated completely due to the diversity of cell types in the whole colon. For the first time, this research revealed the RNA profile alternations of IEL in intestinal inflammation by studying the changes of IEL-related mRNA and lncRNA profiles in chronic inflammation caused by DSS-induced chronic colitis. It is necessary to emphasize that different IEL subpopulations exhibit significant heterogeneity based on adaptation to the complex environment of the intestinal lumen. Further studies are required to analyze the role and regulatory mechanisms of different IEL subgroups in IBD through single-cell sequencing or spatial transcriptome.

KEGG analysis showed that the NF-kappa B signaling pathway, Natural killer cell mediated cytotoxicity, and Leukocyte transendothelial migration pathway were enriched in both mRNA enrichment and mRNA-lncRNA joint analysis enrichment. The NF-kappa B signaling pathway is a classical inflammatory pathway. Previous studies have shown a range of phenotypic and functional similarities between IEL and NK cells [16]. The Leukocyte transendothelial migration pathway was the most significantly upregulated pathway, suggesting a potent recruitment and infiltration ability of IEL in colitis. As previously mentioned, IELs in chronic colitis demonstrate alterations in multiple metabolic pathways, including Tryptophan metabolism, Sulfur metabolism, Histidine metabolism, Nitrogen metabolism et al. Numerous studies have confirmed that tryptophan and its metabolites play a central role in intestinal immunity via the AhR receptor [66, 67]; Sulfur metabolism [68], histidine metabolism [69], and Nitrogen metabolism [70, 71] has also been reported to be involved in intestinal inflammation and immunity. However, the effect of other pathways in immunity remains to be further studied.

High-sugar diets directly contribute to increased endogenous insulin. In line with our results, several studies have shown that high-sugar diets significantly exacerbate experimental colitis based on microbiota [43, 72, 73]. However, inhibition of the insulin pathway also exacerbated colitis: streptozocin-induced Type1 Diabetes mice (endogenous insulin deficiency) exhibited significant exacerbation of colitis and hyperglycemia (relative inhibition of the insulin pathway) is a direct cause of disruption of the intestinal barrier and exacerbation of enteric infections [74]. As insulin can hardly cross the intestinal barrier, delivery by enema or nano-loaded drugs can achieve precise drug targeting to the intestinal mucosa. On the other hand, high-sugar diet is an important feature of “Western diet”, which showed exacerbating effect on cardiovascular, cancer, and autoimmune diseases. Insulin may participate in the pathological process of above diseases as one mechanism of Western diet.

Exacerbation of colitis by INSR appears to contradict with the downregulation of INSR in mucosal T-cells. We believe that the downregulation of INSR in mucosal T cells may act as a protective mechanism in colitis. Pathogenic and protective factors are often upregulated simultaneously in IBD, and the alternating dominance of pathogenic and protective factors leads to the flare-up and remission of IBD [2]. For example, both numbers and function of Treg are increased in colitis, although Treg shows a fundamental effect on suppressing autoimmunity and relieving colitis. In addition, equal expressions of INSR in intestinal epithelial cell and LP cells without lymphocytes was detected in control and DSS group. A reasonable assumption is that the downregulation of INSR by T cells facilitates the reduction of inflammation, while the equal expressions of INSR by epithelial cells and LP cells without lymphocytes facilitates intestinal barrier repair and tissue remodeling. However, the mechanisms that control the distinct changes of INSR in different cell types are required to be further investigated.

Consistent with Zhang et al. this study showed that INSR activation exacerbated experimental colitis [26]. However, Yassin reported that rectal insulin instillation alleviates colitis and colorectal carcinogenesis [51]. The difference may be attributed to the different timing of rectal insulin instillation. In this study, the administration was clustered in the inflammatory phase, while the previous study mainly administrated in the remission phase, suggesting that different time of rectal insulin instillation administration may exhibit diverse effects. Expression of INSR in colonic T cells is higher than in colonic epithelial cells, suggesting that rectal insulin instillation had a stronger effect on T cells than epithelial cells. Therefore we hypothesize that, during the acute inflammatory phase, accompanied by massive effector T cells infiltration, rectal insulin instillation promotes TRM differentiation, especially CD4 TRM, which contributes to the exacerbated inflammation and chronic inflammation formation; while in the remission phase, with the receding of effector T cells and relatively increased expression of INSR in intestinal epithelial cells, the effect of rectal insulin instillation in exacerbating colitis diminishes and instead manifests in promotion of intestinal epithelial cells proliferation and intestinal barrier repair.

Current IBD therapeutic agents show an obvious defect, high risk of relapse in long-term control due to individual differences. Our study found that rectal insulin instillation exacerbated colitis through the regulatory effect of EZH2 on TRM, suggesting that both insulin inhibitor and EZH2 inhibitor could be potential drugs for IBD treatment. When patients were diagnosed with IBD by endoscopic biopsy, changes in EZH2 and INSR expression could be detected by immunohistochemistry or other methods. Patients with EZH2 and INSR alterations could be treated with INSR/EZH2 targeted therapy. Second, it was detected that rectal insulin instillation in colitis-active phase significantly exacerbated colitis, suggesting that rectal insulin instillation should be forbidden during acute-colitis phase of IBD, which complements the deficiencies of previous studies. Last but not least, considering the lack of specific INSR inhibitor and the colitis-exacerbating effect of co-inhibitors, the development of insulin-specific inhibitors is urgently needed to study the INSR-targeted therapy for patients with IBD or other autoimmunity disease.

Numerous studies have demonstrated the antitumor effects of EZH2 inhibition therapy in vivo and immunosuppressed mice [29]. However, both clinical and animal studies have shown that the anti-tumor effect of EZH2 inhibition therapy was significantly reduced in human or normal mice [75, 76]. A major difference in tumor growth in vivo and in vitro comes from immune system. In recent years, the role of TRMs in tumor immunity has been continuously defined [77, 78]. Tissue-resident T cells were shown to be early responders to cancer immunotherapy and potential cells for adoptive immunotherapy against cancer in latest studies [78, 79]. Our study demonstrates the critical regulatory role of EZH2 on TRM. Thus, the use of EZH2 inhibitors in vivo suppressed tumor growth, whereas TRM production and tumor immunity were inhibited at the same time, ultimately leading to the loss of tumor suppression. In the study of EZH2 on tumor therapy, our study suggests that specific inhibitors that can reduce its TRM-inhibitory effect should be further developed, or act precisely on tumor through targeted delivery systems such as nanomaterials. More broadly, since immune cells are also rapidly-proliferating cells, they tend to share certain same pathways with tumor cells, such as MYC [80]. Therefore, in addition to tumor-suppressive effects, more attention should be paid to the immune-regulatory effects in the study of tumor-targeting drugs.

Inevitably, there are some limitations to this research. Although we used human databases, we were unavailable to validate the specific up-regulation of INSR in colonic mucosal lymphocytes in IBD specimens due to the constraints of funding and human ethical. As for the specific mechanisms underlie the regulation of EZH2 in TRM differentiation, this study was not able to provide a clear explanation due to time and financial constraints. EZH2 may be engaged to TRM differentiation through the regulation of TRM core transcription factors such as Hobit, Blimp in a way of histone modification or non-histone modification, which will be further elaborated in subsequent research.

## Conclusion

In conclusion, activation of the insulin pathway upregulates EZH2 expression in intestinal mucosal T cells during inflammation, which in turn promoted TRM differentiation and disrupted subgroups of IELs and inflammatory cytokines, ultimately exacerbated colitis. This study suggests that the intestinal insulin pathway exacerbate colitis during the inflammatory phase; EZH2 is one of the key downstream mechanisms in the regulation of intestinal immunity by insulin pathway; CD4^+^TRM shows a key role in the induction of chronic inflammation and were regulated by insulin pathway and EZH2; the diminished anti-tumor efficacy of EZH2 inhibition therapy in vivo may be due to its suppressive effects on TRM.

### Supplementary Information

**Additional file 1: Table S1.** Top10 down-regulated DEmRNA in joint analysis and Top30 up-regulated lncRNA in joint analysis.** Figure S1.** Construction of DSS induced chronic colitis and purity of IEL.** Figure S2.** Verification of mRNA and lncRNA.** Figure S3.** Protein-protein interaction and ceRNA network of INSR.** Figure S4.** Stimulation of endogenous insulin secretion in different methods shows distinct effects on DSS induced colitis.** Figure S5.** No significant changes of expression of TNF-a,IL-17 and Foxp3 were detected in MLMs after rectal insulin instillation.** Figure S6.** Rectal GSK1904529A instillation exacerbates DSS induced colitis.** Figure S7.** TNF-α and IL-17 secreting T cells highly express CD69 both in IELs and LPLs.

## Data Availability

The datasets of Figs. 1, 2a–f and 5a are available in the GEO database. The other datasets analyzed during the current study are available from the corresponding author on reasonable request.

## Abbreviations

IBD: Inflammatory bowel diseases
ILCs: Innate lymphoid cells
TRM: Tissue resident memory T cells
IELs: Intestinal intraepithelial lymphocytes
INSR: Insulin receptor
EZH2: Enhancer of zeste homolog 2
PRC2: Polycomb repressive complex 2
H3K27me3: Trimethylation of Lys-27 in histone 3
Treg: Regulatory T cell
LPLs: Lamina propria lymphocytes
LP: Lamina propria
CBA: Cytometric bead array
lncRNA: Long no coding RNA
DEmRNA: Differentially expressing mRNA
DElncRNA: Differentially expressed lncRNA
DAI: Disease activity index

## References

[1] Kaplan GG (2015). The global burden of IBD: from 2015 to 2025. Nat Rev Gastroenterol Hepatol.

[2] de Souza HS, Fiocchi C (2016). Immunopathogenesis of IBD: current state of the art. Nat Rev Gastroenterol Hepatol.

[3] Cho JH (2008). The genetics and immunopathogenesis of inflammatory bowel disease. Nat Rev Immunol.

[4] Murray A, Nguyen TM, Parker CE, Feagan BG, MacDonald JK (2020). Oral 5-aminosalicylic acid for maintenance of remission in ulcerative colitis. Cochrane Database Syst Rev.

[5] Faubion WA, Loftus EV, Harmsen WS, Zinsmeister AR, Sandborn WJ (2001). The natural history of corticosteroid therapy for inflammatory bowel disease: a population-based study. Gastroenterology.

[6] Rutgeerts P, Sandborn WJ, Feagan BG (2005). Infliximab for induction and maintenance therapy for ulcerative colitis. N Engl J Med.

[7] Lee JY, Hall JA, Kroehling L (2020). Serum amyloid A proteins induce pathogenic Th17 cells and promote inflammatory disease. Cell.

[8] Negi S, Saini S, Tandel N, Sahu K, Mishra RPN, Tyagi RK (2021). Translating treg therapy for inflammatory bowel disease in humanized mice. Cells.

[9] Zeng B, Shi S, Ashworth G, Dong C, Liu J, Xing F (2019). ILC3 function as a double-edged sword in inflammatory bowel diseases. Cell Death Dis.

[10] Koelink PJ, Bloemendaal FM, Li B (2020). Anti-TNF therapy in IBD exerts its therapeutic effect through macrophage IL-10 signalling. Gut.

[11] Peter A, Szabo MM, Farber DL (2019). Location, location, location: tissue resident memory T cells in mice and humans. Sci Immunol.

[12] Zundler S, Becker E, Spocinska M (2019). Hobit- and Blimp-1-driven CD4(+) tissue-resident memory T cells control chronic intestinal inflammation. Nat Immunol.

[13] Hegazy AN, West NR, Stubbington MJT (2017). Circulating and tissue-resident CD4(+) T cells with reactivity to intestinal microbiota are abundant in healthy individuals and function is altered during inflammation. Gastroenterology.

[14] Lamb CA, Mansfield JC, Tew GW (2017). alphaEbeta7 integrin identifies subsets of pro-inflammatory colonic CD4+ T lymphocytes in ulcerative colitis. J Crohns Colitis.

[15] Bishu S, El Zaatari M, Hayashi A (2019). CD4+ tissue-resident memory T cells expand and are a major source of mucosal tumour necrosis factor alpha in active Crohn’s disease. J Crohns Colitis.

[16] Cheroutre H, Lambolez F, Mucida D (2011). The light and dark sides of intestinal intraepithelial lymphocytes. Nat Rev Immunol.

[17] Lutter L, van Konijnenburg DPH, Brand EC, Oldenburg B, van Wijk F (2018). The elusive case of human intraepithelial T cells in gut homeostasis and inflammation. Nat Rev Gastroenterol Hepatol.

[18] van Konijnenburg DPH, Reis BS, Pedicord VA, Farache J, Victora GD, Mucida D (2017). Intestinal epithelial and intraepithelial T cell crosstalk mediates a dynamic response to infection. Cell.

[19] Swamy M, Abeler-Dorner L, Chettle J (2015). Intestinal intraepithelial lymphocyte activation promotes innate antiviral resistance. Nat Commun.

[20] Ma H, Qiu Y, Yang H (2021). Intestinal intraepithelial lymphocytes: maintainers of intestinal immune tolerance and regulators of intestinal immunity. J Leukoc Biol.

[21] Ribot JC, Lopes N, Silva-Santos B (2021). gammadelta T cells in tissue physiology and surveillance. Nat Rev Immunol.

[22] Inagaki-Ohara K, Chinen T, Matsuzaki G (2004). Mucosal T cells bearing TCRgammadelta play a protective role in intestinal inflammation. J Immunol.

[23] McDonald BD, Jabri B, Bendelac A (2018). Diverse developmental pathways of intestinal intraepithelial lymphocytes. Nat Rev Immunol.

[24] DePeaux K, Delgoffe GM (2021). Metabolic barriers to cancer immunotherapy. Nat Rev Immunol.

[25] Norton L, Shannon C, Gastaldelli A, DeFronzo RA (2022). Insulin: the master regulator of glucose metabolism. Metabolism.

[26] Zhang D, Jin W, Wu R (2019). High glucose intake exacerbates autoimmunity through reactive-oxygen-species-mediated TGF-beta cytokine activation. Immunity.

[27] Tsai S, Clemente-Casares X, Zhou AC (2018). Insulin receptor-mediated stimulation boosts t cell immunity during inflammation and infection. Cell Metab.

[28] Chen C, Wang Z, Qin Y (2022). Connections between metabolism and epigenetics: mechanisms and novel anti-cancer strategy. Front Pharmacol.

[29] Duan R, Du W, Guo W (2020). EZH2: a novel target for cancer treatment. J Hematol Oncol.

[30] Wan L, Xu K, Wei Y (2018). Phosphorylation of EZH2 by AMPK suppresses PRC2 methyltransferase activity and oncogenic function. Mol Cell.

[31] Kim E, Kim M, Woo DH (2013). Phosphorylation of EZH2 activates STAT3 signaling via STAT3 methylation and promotes tumorigenicity of glioblastoma stem-like cells. Cancer Cell.

[32] Xu K, Wu ZJ, Groner AC (2012). EZH2 oncogenic activity in castration-resistant prostate cancer cells is polycomb-independent. Science.

[33] Gray SM, Amezquita RA, Guan T, Kleinstein SH, Kaech SM (2017). Polycomb repressive complex 2-mediated chromatin repression guides effector CD8(+) T cell terminal differentiation and loss of multipotency. Immunity.

[34] He S, Liu Y, Meng L (2017). Ezh2 phosphorylation state determines its capacity to maintain CD8(+) T memory precursors for antitumor immunity. Nat Commun.

[35] DuPage M, Chopra G, Quiros J (2015). The chromatin-modifying enzyme Ezh2 is critical for the maintenance of regulatory T cell identity after activation. Immunity.

[36] Chen X, Cao G, Wu J (2020). The histone methyltransferase EZH2 primes the early differentiation of follicular helper T cells during acute viral infection. Cell Mol Immunol.

[37] Zhang X, Wang Y, Yuan J (2018). Macrophage/microglial Ezh2 facilitates autoimmune inflammation through inhibition of Socs3. J Exp Med.

[38] Zhou J, Huang S, Wang Z (2019). Targeting EZH2 histone methyltransferase activity alleviates experimental intestinal inflammation. Nat Commun.

[39] He J, Song Y, Li G (2019). Fbxw7 increases CCL2/7 in CX3CR1hi macrophages to promote intestinal inflammation. J Clin Invest.

[40] Liu Y, Peng J, Sun T (2017). Epithelial EZH2 serves as an epigenetic determinant in experimental colitis by inhibiting TNFalpha-mediated inflammation and apoptosis. Proc Natl Acad Sci USA.

[41] Wirtz S, Popp V, Kindermann M (2017). Chemically induced mouse models of acute and chronic intestinal inflammation. Nat Protoc.

[42] Fischer HJ, Sie C, Schumann E (2017). The insulin receptor plays a critical role in T cell function and adaptive immunity. J Immunol.

[43] Khan S, Waliullah S, Godfrey V (2020). Dietary simple sugars alter microbial ecology in the gut and promote colitis in mice. Sci Transl Med.

[44] Sullivan ZA, Khoury-Hanold W, Lim J (2021). gammadelta T cells regulate the intestinal response to nutrient sensing. Science.

[45] Borys SM, Bag AK, Brossay L, Adeegbe DO (2022). The yin and yang of targeting KLRG1(+) tregs and effector cells. Front Immunol.

[46] Meinicke H, Bremser A, Brack M, Schrenk K, Pircher H, Izcue A (2017). KLRG1 impairs regulatory T-cell competitive fitness in the gut. Immunology.

[47] Belfiore A, Malaguarnera R, Vella V (2017). Insulin receptor isoforms in physiology and disease: an updated view. Endocr Rev.

[48] Sabbatini P, Rowand JL, Groy A (2009). Antitumor activity of GSK1904529A, a small-molecule inhibitor of the insulin-like growth factor-I receptor tyrosine kinase. Clin Cancer Res.

[49] Karantanos T, Chistofides A, Barhdan K, Li L, Boussiotis VA (2016). Regulation of T cell differentiation and function by EZH2. Front Immunol.

[50] Sarmento OF, Svingen PA, Xiong Y (2017). The role of the histone methyltransferase enhancer of zeste homolog 2 (EZH2) in the pathobiological mechanisms underlying inflammatory bowel disease (IBD). J Biol Chem.

[51] Yassin M, Sadowska Z, Tritsaris K (2018). Rectal insulin instillation inhibits inflammation and tumor development in chemically induced colitis. J Crohns Colitis.

[52] McCabe MT, Ott HM, Ganji G (2012). EZH2 inhibition as a therapeutic strategy for lymphoma with EZH2-activating mutations. Nature.

[53] Su W, Saravia J, Risch I (2023). CXCR6 orchestrates brain CD8+ T cell residency and limits mouse Alzheimer’s disease pathology. Nat Immunol.

[54] Ogongo P, Tezera LB, Ardain A (2021). Tissue-resident-like CD4+ T cells secreting IL-17 control Mycobacterium tuberculosis in the human lung. J Clin Invest.

[55] Han J, Zhao Y, Shirai K (2021). Resident and circulating memory T cells persist for years in melanoma patients with durable responses to immunotherapy. Nat Cancer.

[56] Kaech SM, Cui W (2012). Transcriptional control of effector and memory CD8+ T cell differentiation. Nat Rev Immunol.

[57] Andrejeva G, Rathmell JC (2017). Similarities and distinctions of cancer and immune metabolism in inflammation and tumors. Cell Metab.

[58] Shyer JA, Flavell RA, Bailis W (2020). Metabolic signaling in T cells. Cell Res.

[59] Kumar A, Chamoto K (2021). Immune metabolism in PD-1 blockade-based cancer immunotherapy. Int Immunol.

[60] Chan JD, Lai J, Slaney CY, Kallies A, Beavis PA, Darcy PK (2021). Cellular networks controlling T cell persistence in adoptive cell therapy. Nat Rev Immunol.

[61] Wahl DR, Byersdorfer CA, Ferrara JL, Opipari AW, Glick GD (2012). Distinct metabolic programs in activated T cells: opportunities for selective immunomodulation. Immunol Rev.

[62] Makowski L, Chaib M, Rathmell JC (2020). Immunometabolism: from basic mechanisms to translation. Immunol Rev.

[63] Chang CH, Curtis JD, Maggi LB (2013). Posttranscriptional control of T cell effector function by aerobic glycolysis. Cell.

[64] Menk AV, Scharping NE, Moreci RS (2018). Early TCR signaling induces rapid aerobic glycolysis enabling distinct acute T cell effector functions. Cell Rep.

[65] Ahmed F, Ho SG, Samantasinghar A (2022). Drug repurposing in psoriasis, performed by reversal of disease-associated gene expression profiles. Comput Struct Biotechnol J.

[66] Agus A, Planchais J, Sokol H (2018). Gut microbiota regulation of tryptophan metabolism in health and disease. Cell Host Microbe.

[67] Pernomian L, Duarte-Silva M, de Barros Cardoso CR (2020). The aryl hydrocarbon receptor (AHR) as a potential target for the control of intestinal inflammation: insights from an immune and bacteria sensor receptor. Clin Rev Allergy Immunol.

[68] Metwaly A, Dunkel A, Waldschmitt N (2020). Integrated microbiota and metabolite profiles link Crohn’s disease to sulfur metabolism. Nat Commun.

[69] Wu J, Wu Y, Feng W (2022). Role of microbial metabolites of histidine in the development of colitis. Mol Nutr Food Res.

[70] Guzik TJ, Korbut R, Adamek-Guzik T (2003). Nitric oxide and superoxide in inflammation and immune regulation. J Physiol Pharmacol.

[71] Mu K, Yu S, Kitts DD (2019). The role of nitric oxide in regulating intestinal redox status and intestinal epithelial cell functionality. Int J Mol Sci.

[72] Laffin M, Fedorak R, Zalasky A (2019). A high-sugar diet rapidly enhances susceptibility to colitis via depletion of luminal short-chain fatty acids in mice. Sci Rep.

[73] Kovatcheva-Datchary P, Nilsson A, Akrami R (2015). Dietary fiber-induced improvement in glucose metabolism is associated with increased abundance of prevotella. Cell Metab.

[74] Thaiss CA, Levy M, Grosheva I (2018). Hyperglycemia drives intestinal barrier dysfunction and risk for enteric infection. Science.

[75] Yap TA, Winter JN, Giulino-Roth L (2019). Phase I study of the novel enhancer of zeste homolog 2 (EZH2) inhibitor GSK2816126 in patients with advanced hematologic and solid tumors. Clin Cancer Res.

[76] Huang S, Wang Z, Zhou J (2019). EZH2 inhibitor GSK126 suppresses antitumor immunity by driving production of myeloid-derived suppressor cells. Cancer Res.

[77] Okla K, Farber DL, Zou W (2021). Tissue-resident memory T cells in tumor immunity and immunotherapy. J Exp Med.

[78] Anadon CM, Yu X, Hanggi K (2022). Ovarian cancer immunogenicity is governed by a narrow subset of progenitor tissue-resident memory T cells. Cancer Cell.

[79] Beumer-Chuwonpad A, Taggenbrock R, Ngo TA, van Gisbergen K (2021). The potential of tissue-resident memory T cells for adoptive immunotherapy against cancer. Cells.

[80] Dar AA, Kim DD, Gordon SM (2023). c-Myc uses Cul4b to preserve genome integrity and promote antiviral CD8+ T cell immunity. Nat Commun.

