# Supplementary material for: Activation of mucosal insulin receptor exacerbates intestinal inflammation by promoting tissue resident memory T cells differentiation through EZH2
Source: J Transl Med. 2024 Jan 19;22:78. doi: 10.1186/s12967-023-04789-x (PMC10797971; doi:10.1186/s12967-023-04789-x)
Supplement: Supplementary file 1 — Additional file 1: Table S1. Top10 down-regulated DEmRNA in joint analysis and Top30 up-regulated lncRNA in joint analysis. Figure S1. Construction of DSS induced chronic colitis and purity of IEL. Figure S2. Verification of mRNA and lncRNA. Figure S3. Protein-protein interaction and ceRNA network of INSR. Figure S4. Stimulation of endogenous insulin secretion in different methods shows distinct effects on DSS induced colitis. Figure S5. No significant changes of expression of TNF-a,IL-17 and Foxp3 were detected in MLMs after rectal insulin instillation. Figure S6. Rectal GSK1904529A instillation exacerbates DSS induced colitis. Figure S7. TNF-α and IL-17 secreting T cells highly express CD69 both in IELs and LPLs. [file 12967_2023_4789_MOESM1_ESM.doc]

**Tables**


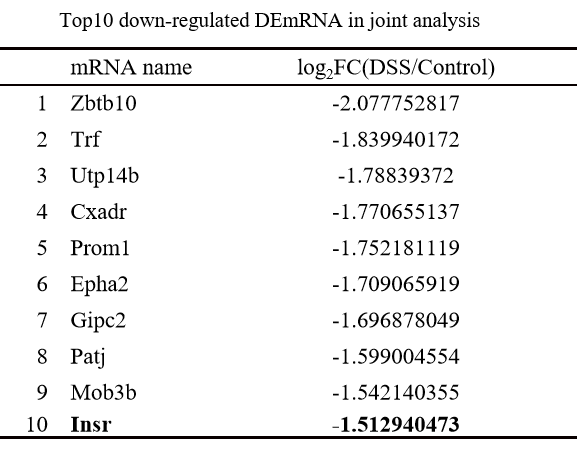


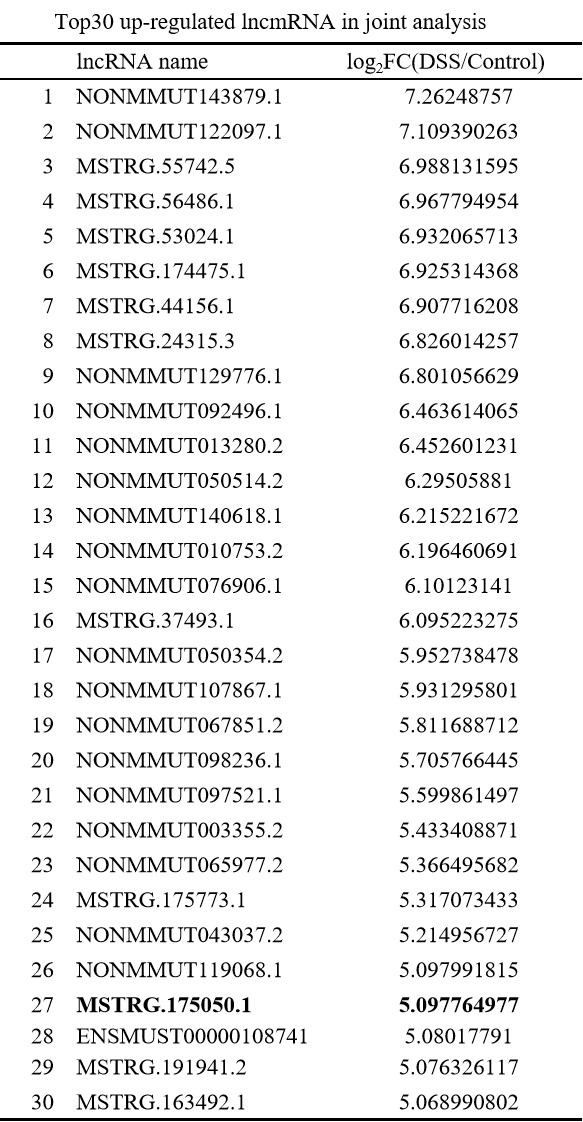


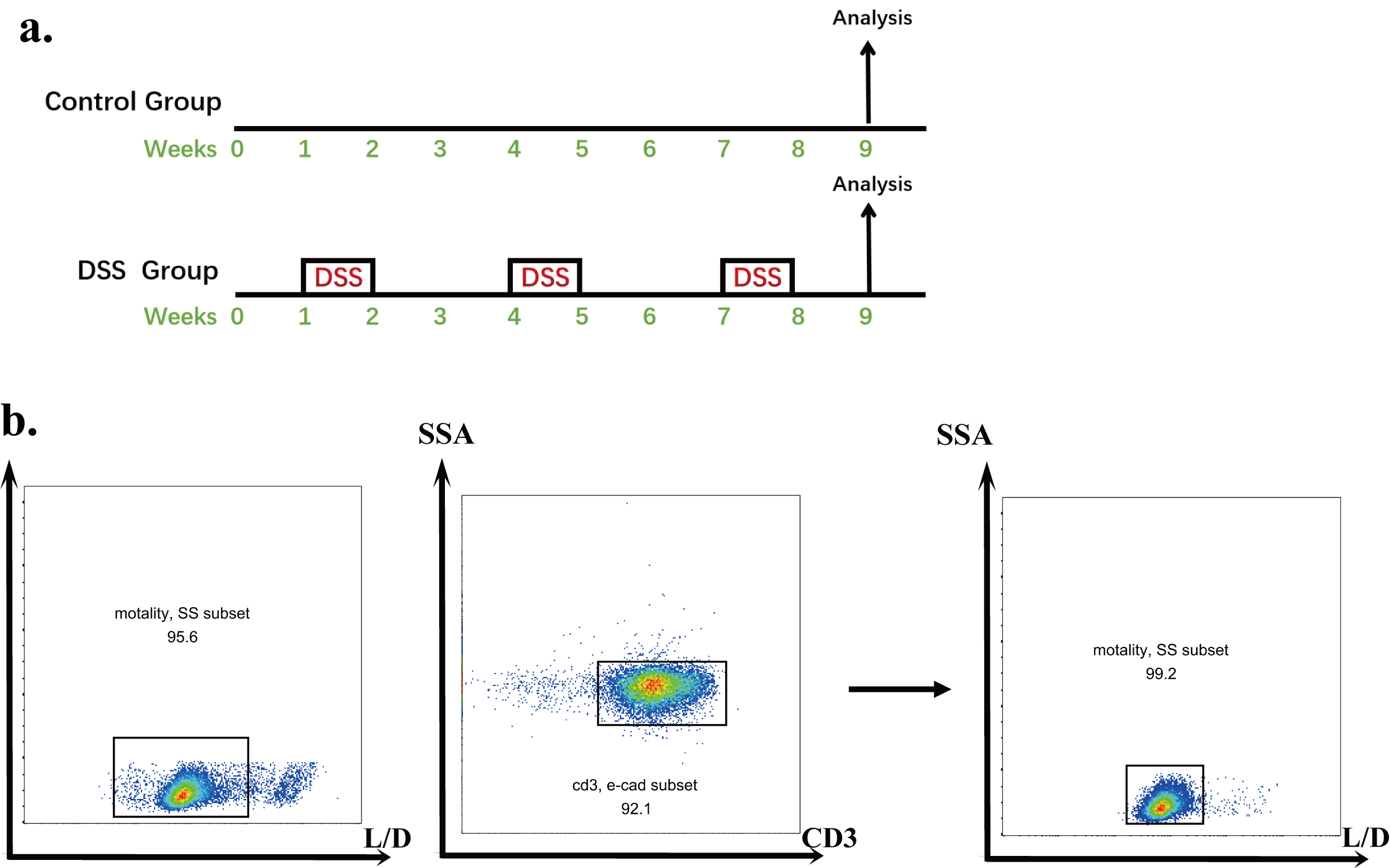


**Figure S1 Construction of DSS induced chronic colitis and purity of IEL**

**a.**A animal model schematic outline of DSS induced chronic colitis

**b.**purity of IEL were showed by percentage of living cell and CD3+E-cadherin -cells.

**
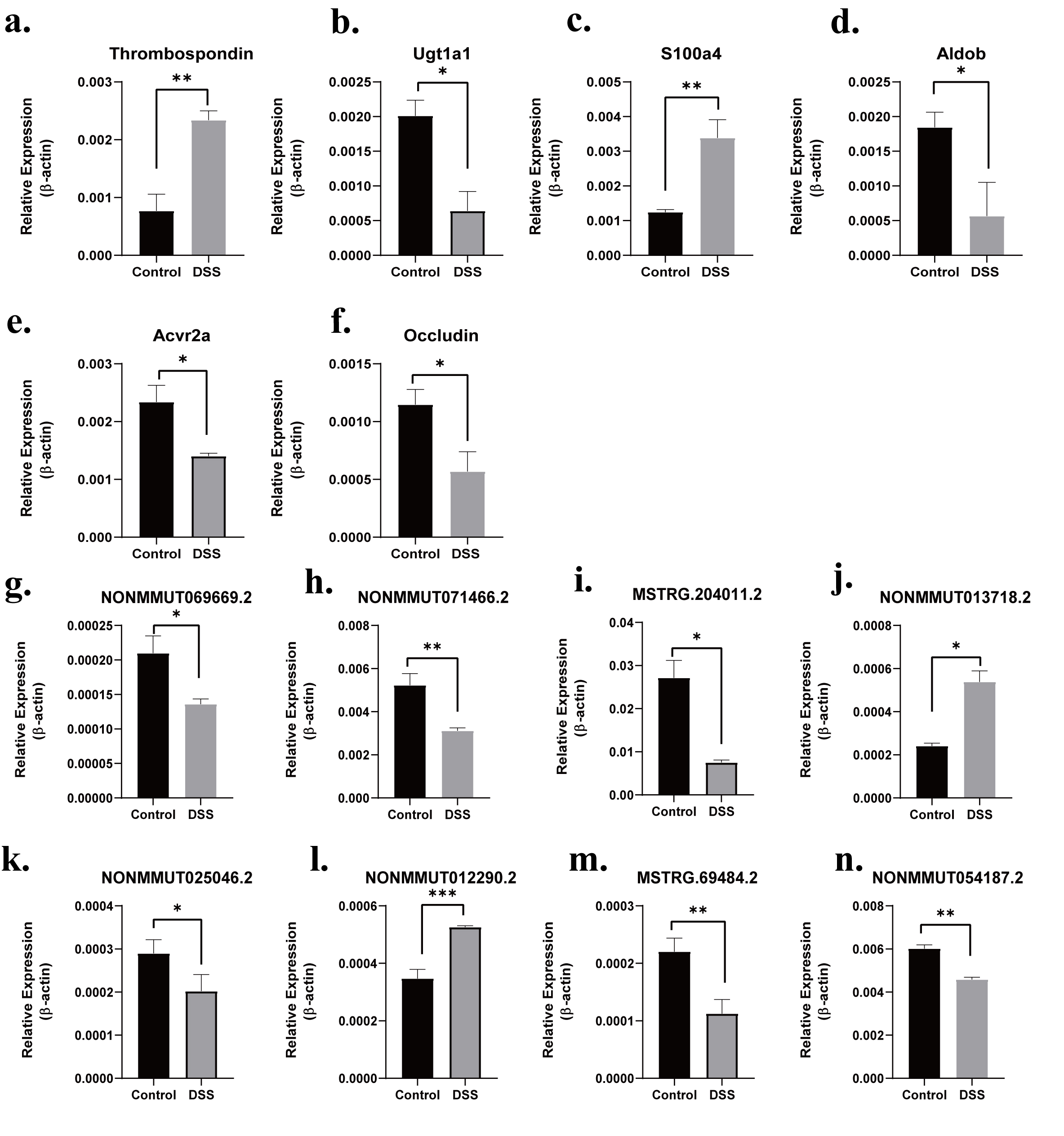
**

**Figure S2 Verification of mRNA and lncRNA**

a.6 mRNA were choosen randomly for verification

b.8 lncRNA were choosen randomly for verification

**
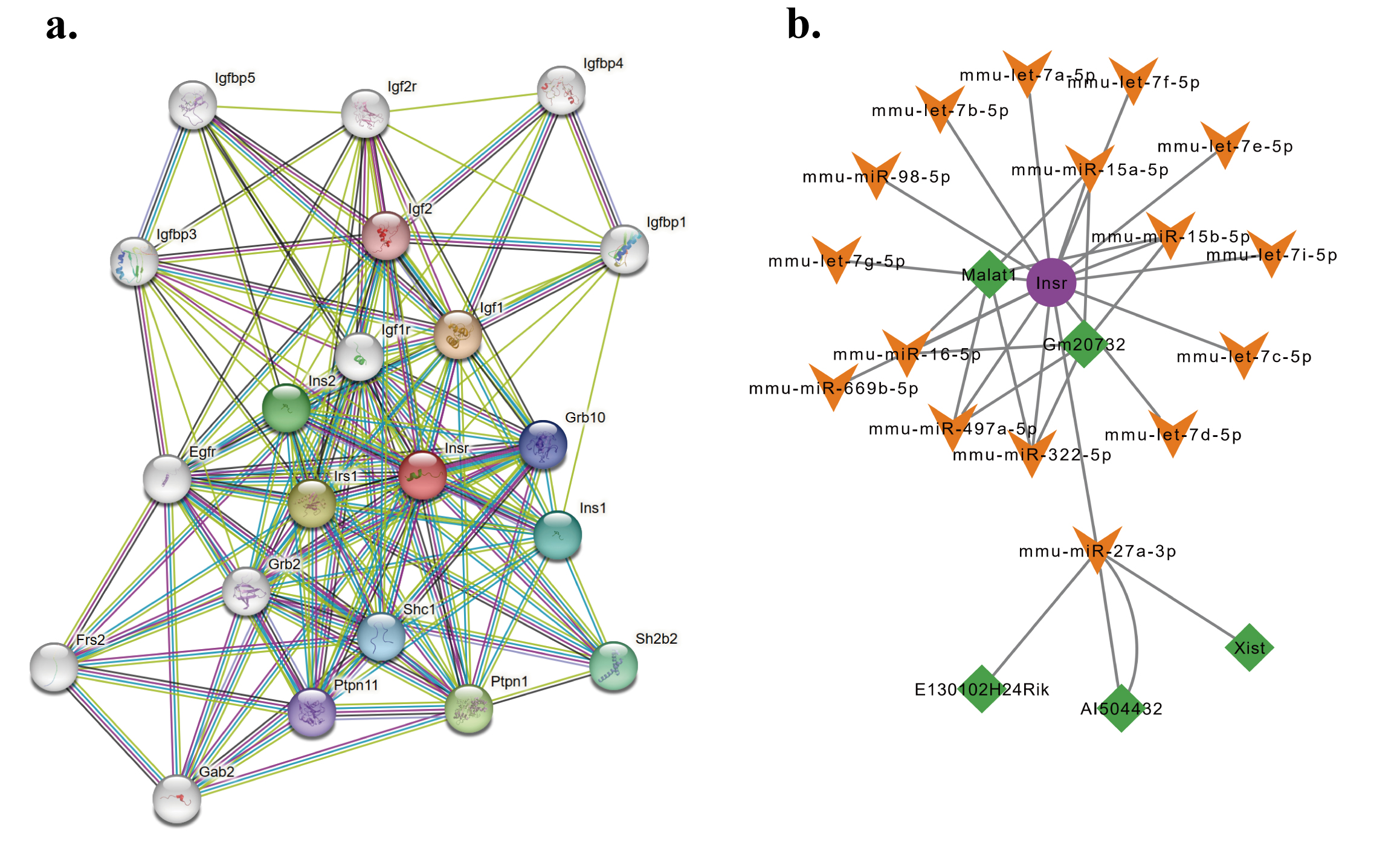
**

**Figure S3 Protein-protein interaction and ceRNA network of INSR**

a.Protein-protein interaction of INSR

b.ceRNA network of INSR

**
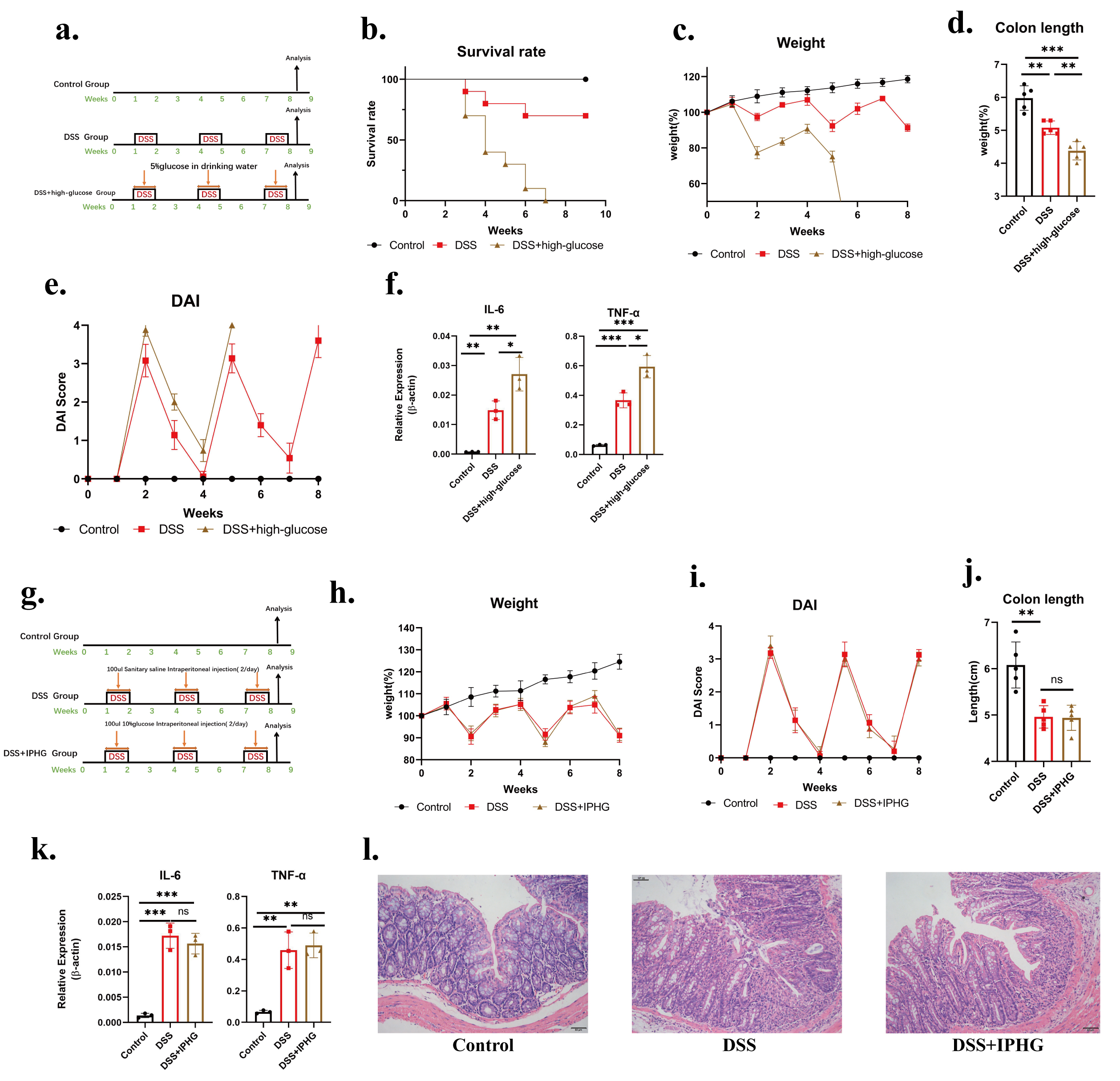
**

**Figure S4 Stimulation of endogenous insulin secretion in different methods shows distinct effects on DSS induced colitis**

1. a animal model schematic outline of Control group,DSS group and DSS+high-glucose group.Five mice at least per group were analyzed.

b-e. Survival rates,weight change,colon length and DAI score of Control group,DSS group and DSS+high-glucose group.

f. mRNA expression of IL-6 and TNF-ɑ in Control group,DSS group and DSS+high-glucose group were analyzed by qPCR.

g. a animal model schematic outline of Control group,DSS group and DSS+IPHG group.Five mice at least per group were analyzed.

h-j. weight change,DAI score and colon length of Control group,DSS group and DSS+IPHG group.

k. mRNA expression of IL-6 and TNF-ɑ in Control group,DSS group and DSS+IPHG group were analyzed by qPCR.

l. representative histological sections of the colon from Control group,DSS group and DSS+IPHG group. Sections were stained with hematoxylin and eosin.

**
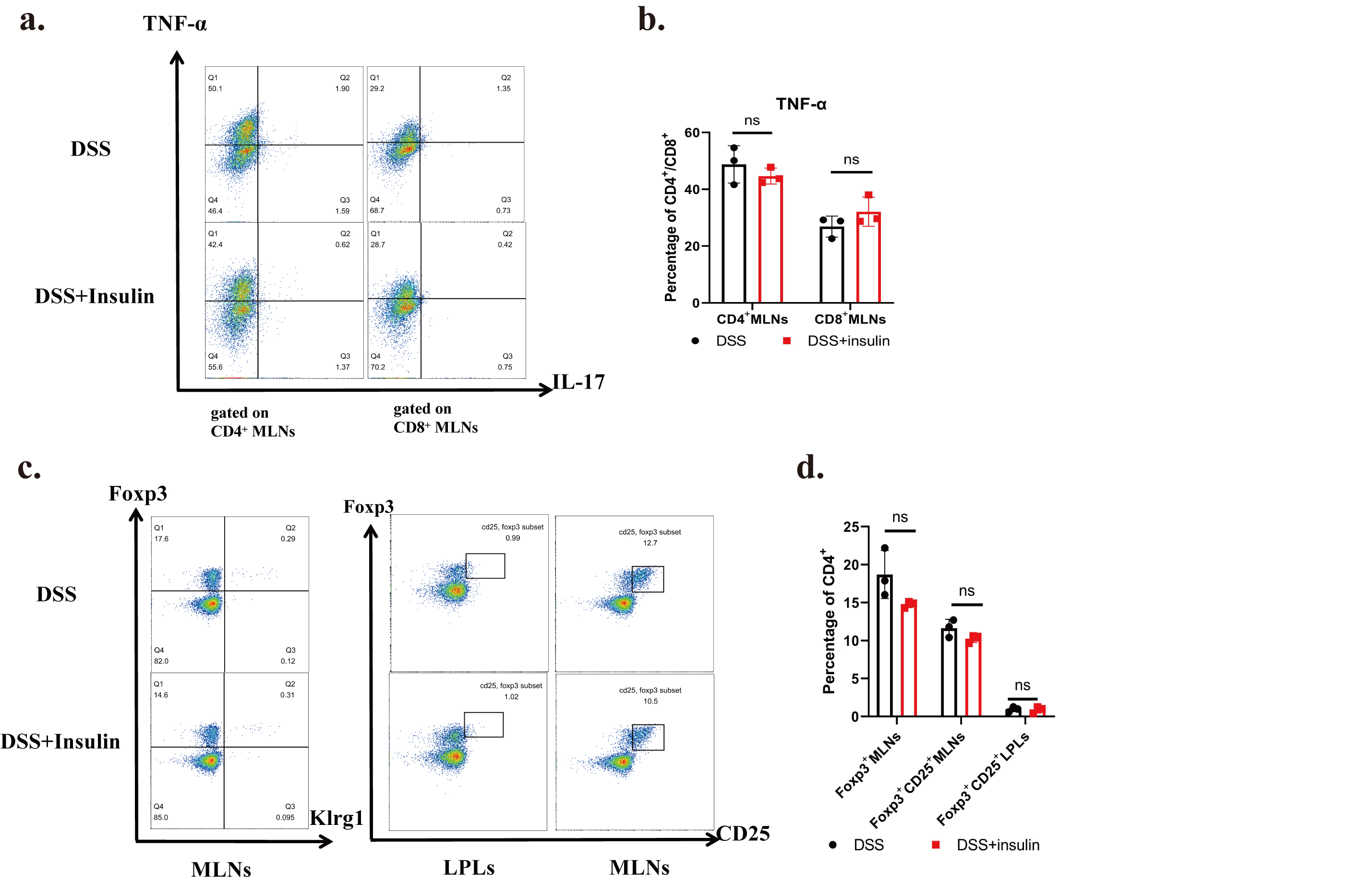
**

**Figure S5 No significant changes of expression of TNF-a,IL-17 and Foxp3 were detected in MLMs after insulin enucleation**

**a.**Representative plots of Intracellular-staining of TNF-ɑ, IL-17 in CD4+ and CD8αβ+ MLNs from DSS group and DSS+insulin(12U/Kg) group.CD4+ and CD8αβ+ MLNs were gated on CD3+live cells.

**b.**Percentages of TNF-ɑ,IL-17 in colonic CD4+ and CD8αβ+ MLNs of DSS group and DSS+insulin(12U/Kg) group. The findings of three pooled independent experiments are shown.

**c.**Representative plots of Foxp3,Klrg1 and CD25 TNF-ɑ, IL-17 in CD4+ and CD8αβ+ MLNs or LPLs from DSS group and DSS+insulin(12U/Kg) group.CD4+ and CD8αβ+ MLNs were gated on CD3+live cells.

**d.**Percentages of Foxp3+MLNs, Foxp3+CD25+MLNs and Foxp3+CD25+LPLs of DSS group and DSS+insulin(12U/Kg) group. The findings of three pooled independent experiments are shown.

**
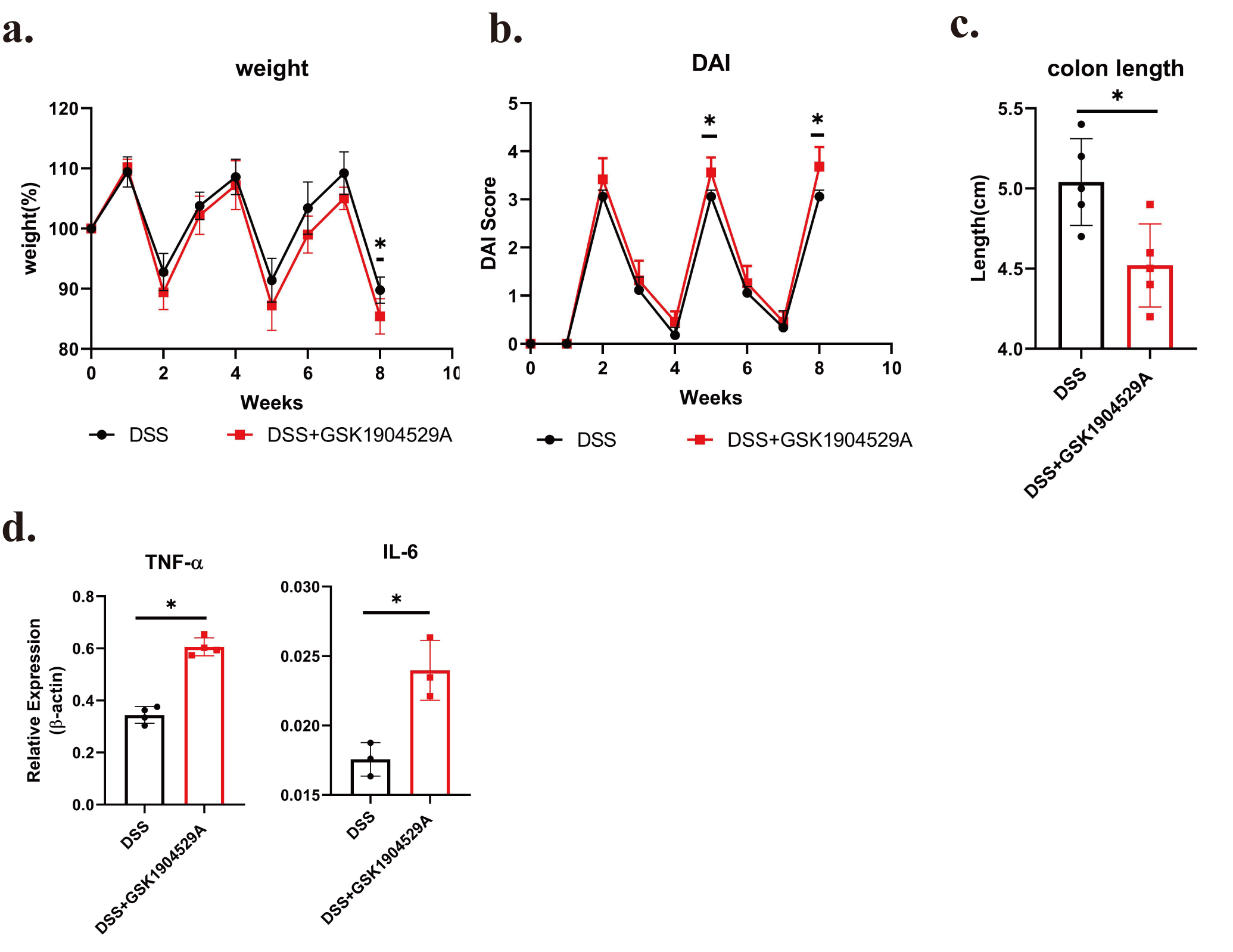
**

**Figure S6 rectal GSK1904529A instillation exacerbates DSS induced colitis**

**a-c.**weight change,DAI score and colon length of DSS group and DSS+GSK1904529A group.

**b.**mRNA expression of IL-6 and TNF-ɑ in DSS group and DSS+GSK1904529A group were analyzed by qPCR.

**
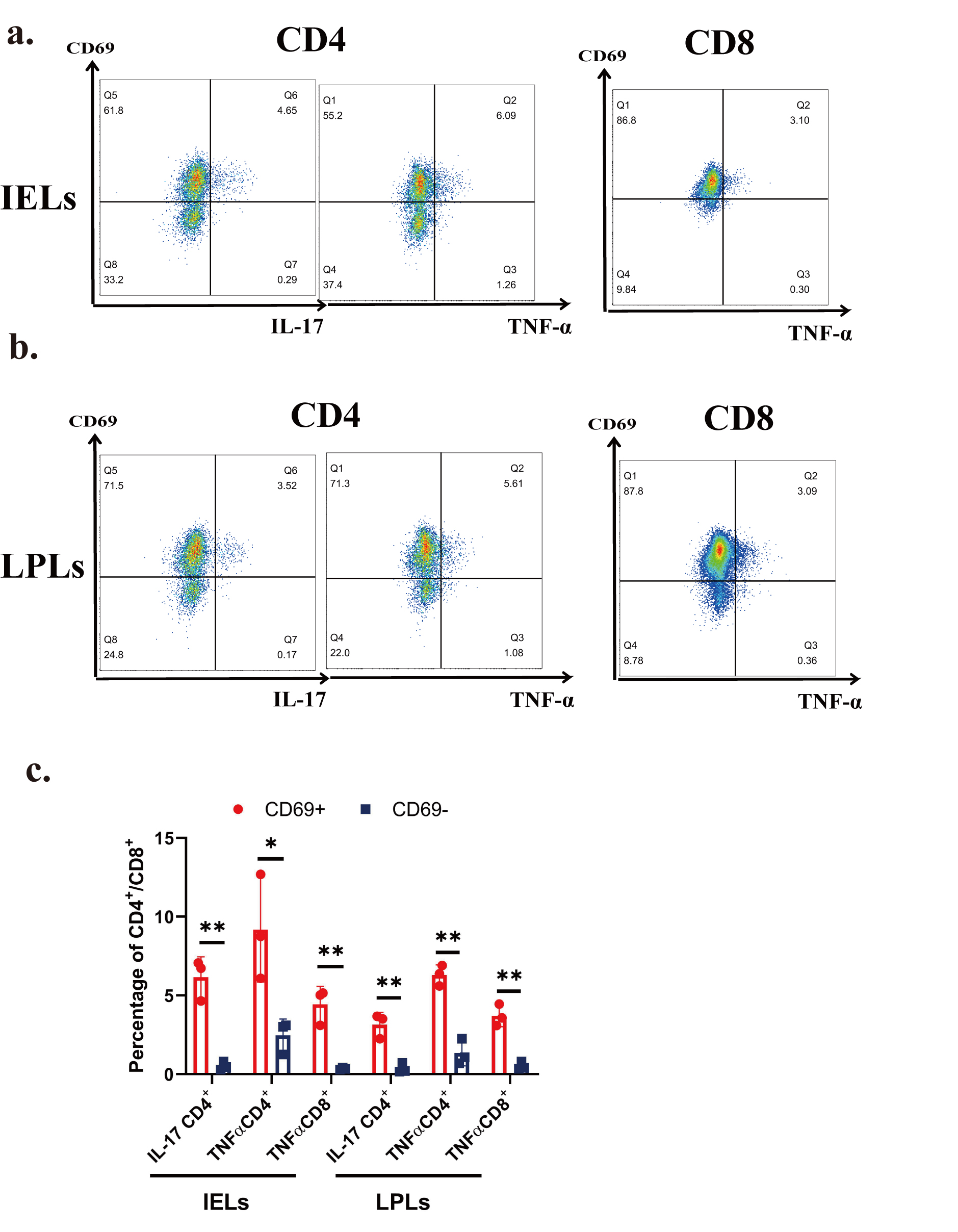
**

**Figure S7 TNF-α and IL-17 secreting T cells highly express CD69 both in IELs and LPLs**

**a.**Representative plots of TNF-ɑ, IL-17 and CD69 in CD4+ and CD8αβ+ IELs from DSS+insulin(12U/Kg) group.CD4+ and CD8αβ+IELs were gated on CD3+live cells

**b.**Representative plots of TNF-ɑ, IL-17 and CD69 in CD4+ and CD8αβ+ LPLs from DSS+insulin(12U/Kg) group.CD4+ and CD8αβ+LPLs were gated on CD3+live cells

**c.**Percentages of TNF-a+,IL-17+in CD69+ or CD69- colonic T cells. The findings of three pooled independent experiments are shown.
